# Real-World Setting of Efficacy and Safety of 3 Years of Rifaximin Administration in Japanese Patients with Hepatic Encephalopathy: A Multicenter Retrospective Study

**DOI:** 10.3390/jcm14041358

**Published:** 2025-02-18

**Authors:** Hideto Kawaratani, Tadashi Namisaki, Yasuteru Kondo, Ryoji Tatsumi, Naoto Kawabe, Norikazu Tanabe, Akira Sakamaki, Kyoko Hoshikawa, Yoshihito Uchida, Kei Endo, Takumi Kawaguchi, Tsunekazu Oikawa, Yoji Ishizu, Shuhei Hige, Taro Takami, Shuji Terai, Yoshiyuki Ueno, Satoshi Mochida, Kazuhiko Koike, Hitoshi Yoshiji

**Affiliations:** 1 Department of Gastroenterology, Nara Medical University, Kashihara 634-8522, Japan; kawara@naramed-u.ac.jp (H.K.);; 2 Department of Hepatology, Sendai Tokushukai Hospital, Sendai 981-3116, Japan; yasuteru@ebony.plala.or.jp; 3 Department of Gastroenterology, Sapporo Kosei General Hospital, Sapporo 060-0033, Japan; nyantyu_seijin@yahoo.co.jp (R.T.); shuhei.hige@ja-hokkaidoukouseiren.or.jp (S.H.); 4 Department of Gastroenterology and Hepatology, Fujita Health University School of Medicine, Nagoya 454-8509, Japan; kawabe@fujita-hu.ac.jp; 5 Department of Gastroenterology and Hepatology, Graduate School of Medicine, Yamaguchi University, Ube 755-8611, Japan; norikazu@yamaguchi-u.ac.jp (N.T.); t-takami@yamaguchi-u.ac.jp (T.T.); 6 Division of Gastroenterology and Hepatology, Graduate School of Medical and Dental Sciences, Niigata University, Niigata 950-2181, Japan; saka-a@med.niigata-u.ac.jp (A.S.); terais@med.niigata-u.ac.jp (S.T.); 7 Department of Gastroenterology, Yamagata University Faculty of Medicine, Yamagata 990-9585, Japan; to.kyoko@med.id.yamagata-u.ac.jp (K.H.); y-ueno@med.id.yamagata-u.ac.jp (Y.U.); 8 Department of Gastroenterology & Hepatology, Faculty of Medicine, Saitama Medical University, Saitama 350-0495, Japan; uchida.yoshihito@1972.saitama-med.ac.jp (Y.U.); smochida@saitama-med.ac.jp (S.M.); 9 Division of Hepatology, Department of Internal Medicine, Iwate Medical University School of Medicine, Yahaba 028-3695, Japan; keiendo@iwate-med.ac.jp; 10 Division of Gastroenterology, Department of Medicine, Kurume University School of Medicine, Kurume 830-0011, Japan; takumi@med.kurume-u.ac.jp; 11 Division of Gastroenterology and Hepatology, Department of Internal Medicine, The Jikei University School of Medicine, Tokyo 105-8461, Japan; 12 Department of Gastroenterology and Hepatology, Nagoya University Graduate School of Medicine, Nagoya 466-8550, Japan; y-ishizu@med.nagoya-u.ac.jp; 13 Kanto Central Hospital, Tokyo 158-8531, Japan; kkoike.tky@gmail.com

**Keywords:** hepatic encephalopathy, Japanese, long-term administration, multicentered, rifaximin

## Abstract

**Background/Objectives:** Rifaximin is a therapeutic agent for patients with hepatic encephalopathy (HE); however, there is little data on the effects of its long-term (>1 year) administration in Japanese patients with cirrhosis. The effects and safety of 3-year rifaximin treatment on HE was investigated in Japan. **Methods:** A total of 190 Japanese patients with cirrhosis who were continuously administered rifaximin for more than 1 year suffered overt or covert HE, which was diagnosed by a physician. Laboratory data were collected at baseline, 3, 6, 12, 18, 24, 30, and 36 months following rifaximin administration. We examined the cumulative overt HE incidences, overall survival rates, and hepatic functional reserves following rifaximin treatment. The occurrence of adverse events was also assessed. **Results:** The levels of ammonia improved significantly after 3 months of rifaximin administration, which continued for 3 years. Serum albumin and prothrombin activity also significantly improved 3 years after initiation of rifaximin treatment. Cumulative overt HE incidences were 12.1%, 19.7%, and 24.9% at 1, 2, and 3 years, respectively. The survival rates following rifaximin treatment were 100%, 88.9%, and 77.8% at 1, 2, and 3 years, respectively. In contrast, renal function and electrolytes did not change following rifaximin administration. Only three (1.6%) patients discontinued rifaximin therapy because of severe diarrhea after 1 year of rifaximin administration. No other serious adverse events were observed. **Conclusions:** Three years of continuous rifaximin (RFX) treatment was both effective and safe for patients with hepatic encephalopathy. Liver function improved and did not worsen during treatment.

## 1. Introduction

Hepatic encephalopathy (HE) is a common complication in patients with liver cirrhosis. It causes varying degrees of neuropsychological and neuromuscular dysfunction [1]. HE is profoundly correlated with poor survival and a substantial reduction in health-related quality of life [2]. HE is defined as a neuropsychiatric syndrome derived from liver dysfunction and portosystemic shunting (PSS), manifesting various neurological or psychiatric disorders [3]. Three recognized types of HE are observed. The first, HE A (acute), is linked to acute liver failure or fulminant hepatitis. The second, HE B (bypass), is associated with portal intrahepatic shunting and occurs in patients without liver disease. The third, HE C (cirrhosis), arises in individuals with liver cirrhosis [4,5,6,7]. In addition, HE is defined as two types of covert and overt encephalopathy by the International Society of Hepatic Encephalopathy and Nitrogen Metabolism (ISHEN) criteria [8]. West Haven criteria (WHC) divide covert HE into minimal and grade I. Covert HE (CHE) is characterized by impaired cognitive function in attention, vigilance, and integrative function; however, obvious clinical manifestation is lacking. Thus, CHE is diagnosed only by neuropsychological testing, neuroimaging, or neurophysiology [5,9]. Overt HE (OHE) is classified into grades II–IV and presents disorders of consciousness and motor activity. After the development of OHE, the prognosis worsens, with a 1-year mortality rate of 64% and 85% within 5 years [10]. CHE occurs in 80% of patients with liver cirrhosis, and covert and overt HE has a poor prognosis and an increased risk of hospitalization [11].

Rifaximin-α (RFX) is a minimally absorbed gut-specific oral antibiotic that is effective against a wide spectrum of organisms, with an extremely low risk of antibiotic resistance and few adverse effects [12]. RFX reduces the recurrence of OHE and is encouraged by the National Institute for Health along with synthesized disaccharides as first-line treatment for HE. In Japan, poorly absorbed antibiotics, such as kanamycin or polymyxin B, have been used for HE treatment [13]; however, they are not approved by the Japanese health insurance system. Rifaximin (Rifxima^®^; ASKA Pharmaceutical, Tokyo, Japan) was approved for HE treatment in November 2016 in Japan [14]; however, data were available up to 12 weeks after the administration [15], and in addition, the long-term efficacy and safety of rifaximin treatment have not been investigated [16,17,18,19]. We previously reported the efficacy and safety of 12 months of RFX administration [20]; however, additional long-term (more than 1 year) data are lacking [21,22]. There are 5 years of data from a single-center study in the UK [23]. As the gut microbiome differs by race [24], RFX effectiveness may vary by population. RFX effectiveness is required to be evaluated in the Japanese cohort. There are 3 years of data from Japan [22]; however, it was from a single center. Therefore, this multicenter retrospective study examined the efficacy and safety of 3 years of RFX treatment in a real-world clinical setting.

## 2. Materials and Methods

### 2.1. Patients

**Figure 1:**
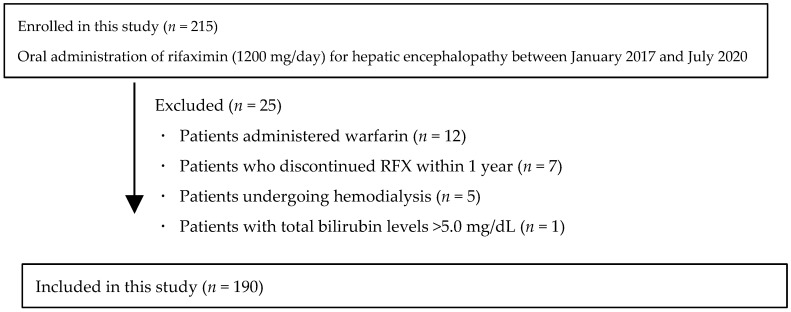
Flowchart of the present study.

This was a retrospective observational cohort study conducted at 12 Japanese centers included in the previous study [20]. A total of 215 patients with liver cirrhosis who were continuously administered 1200 mg of RFX for 1 year in our previous study were enrolled between January 2017 and July 2020. In total, 25 patients who did not visit the hospital or discontinued RFX after less than 1 year were excluded (*n* = 25). A total of 190 patients with cirrhosis who had overt or covert HE and received rifaximin were enrolled (Figure 1). The types of HE were all C-type HE. The enrollment criteria for RFX administration included over HE grade I or if a physician considered it necessary to administer RFX (CHE). The primary endpoint was the effectiveness of 3 years of RFX treatment, and secondary outcomes included the safety of 3 years of RFX treatment and hepatic function reserve, cumulative OHE incidences, and overall survival rates.

### 2.2. Ethics

We performed the study according to the ethical guidelines of the 1975 Declaration of Helsinki and received ethical approval from the independent Ethics Review Committee of all participating institutions. All patients provided written informed consent for blood specimens before study enrollment. The medical history of liver-related complications.

### 2.3. Data Collection

The data were retrospectively collected at each institution. Data were on clinical background, and from blood test results at baseline and at 3, 6, 12, 18, 24, 30, and 36 months after RFX administration. The medical history of liver-related complications was collected. Port systemic shunt (PSS) was assessed by enhanced CT or MRI, and a maximum vessel diameter of 5 mm indicated the presence of PSS. The incidence or recurrence of OHE was defined as HE grade 2 and more severe.

### 2.4. Statistical Analysis

Data are described as the median ± IQR, as appropriate. To compare continuous variables at baseline and after RFX administration at 3, 6, 12, 18, 24, 30, and 36 months, a repeated-measures analysis of variance was used to compare the scores across trials. Dunnett’s post hoc test was used to analyze the data. Overall survival was analyzed using the Kaplan–Meier method, with the event defined as death, which was created with Gray’s test for evaluating cumulative HE occurrence. All statistical analyses were performed with EZR [23]. It is a modified version of R commander, which adds statistical functions often used in biostatistics. *p*-value < 0.05 was considered statistically significant.

## 3. Results

### 3.1. Patient Characteristics

Table 1 shows baseline characteristics. The average age of the patients treated with RFX was 71.0 years, with 61.1% men (116 men and 74 women). The etiology of liver disease involves hepatitis B (19 patients), hepatitis C (33 patients), alcohol-related (57 patients), metabolic dysfunction–associated steatotic liver disease (27 patients), and other causes, including autoimmune hepatitis of the primary biliary cholangitis (54 patients). Forty-seven patients (24.7%) had a history of HE, whereas 14, 154, and 22 patients were Child–Pugh class A, B, and C, respectively. Complications of cirrhosis were associated with esophageal varices (105 patients; 55.3%), PSS (99 patients; 52.1%), splenomegaly (143 patients; 75.3%), ascites (69 patients; 36.3%), no SBP complications, and HCC (61 patients; 32.1%). In the cohort, 21, 27, 8, and 5 patients had stage 1, 2, 3, and 4 HCC, respectively. The results of pretreatment laboratory examinations are listed in Table 1. In total, 62 and 21 patients were treated with lactulose and branched-chain amino acid (BCAA), respectively. Table 2 shows the characteristics of the patients with cirrhosis stratified by sex. Male patients had a significantly higher median body mass index than females (*p* = 0.022). Significantly fewer male than female patients had a portosystemic shunt (*p* < 0.001). Significantly more males than females had a diagnosis of hepatocellular carcinoma (*p* = 0.038). Table 3 shows the characteristics of the patients with cirrhosis stratified by age. There were significantly more females than males aged ≥65 years (*p* = 0.021). There was a significantly higher rate of splenomegaly in patients <65 years than those ≥65 years (*p* = 0.042). Table 4 shows the clinical characteristics of the patients stratified by the type of HE. Patients with OHE were significantly older than those with covert HE (CHE; *p* < 0.001). A significantly higher number of patients with CHE had a history of OHE than those with OHE (*p* < 0.001). Pretreatment laboratory values of patients with hepatic encephalopathy were shown in Table 5.

### 3.2. Efficacy of RFX Treatment

**Figure 2:**
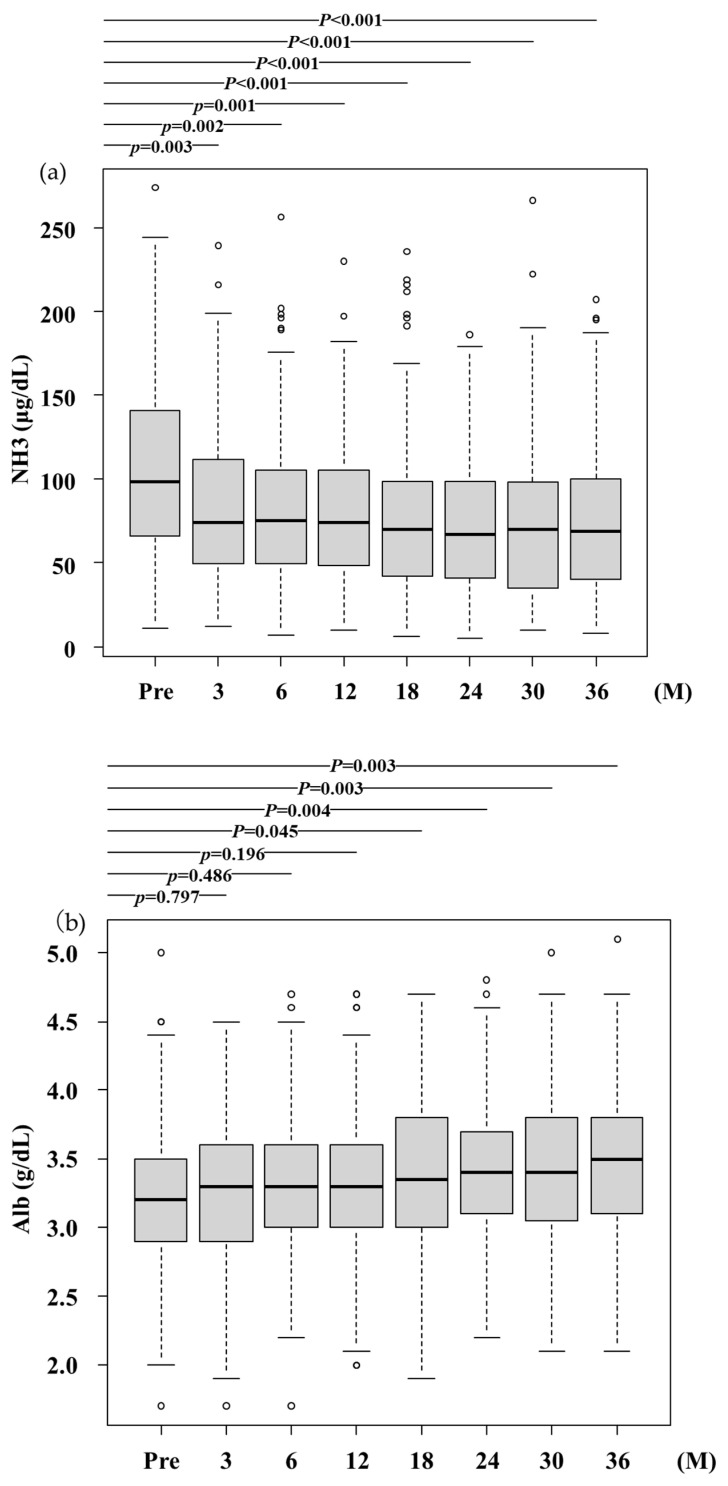

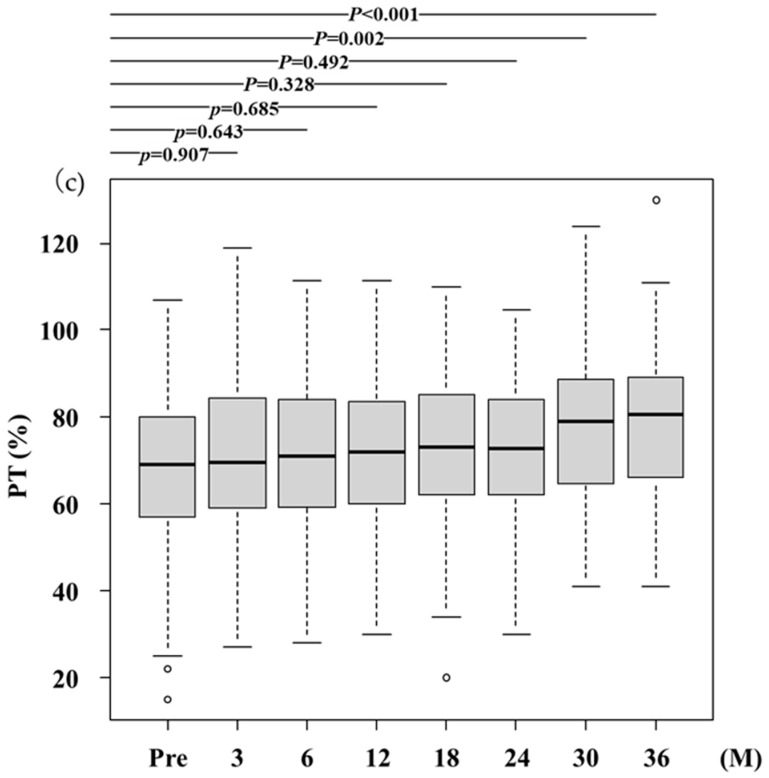
Serum ammonia and albumin levels and prothrombin time (%) after RFX administration. (**a**) NH_3_, Ammonia levels significantly decreased after RFX treatment from 100.0 (66.5–141.5) μg/dL at baseline to 74.0 (49.8–111.3) μg/dL, 75.4 (49.5–105.0) μg/dL, 74.4 (48.5–105.0) μg/dL, 70.2 (42.0–99.0) μg/dL, 67.0 (41.0–99.0) μg/dL, 70.0 (35.3–97.5) μg/dL, and 69.0 (40.0–99.8) μg/dL after 3, 6, 12, 18, 24, 30, and 36 months, respectively. A statistically significant difference was observed between baseline and after 3 months and more. Pre, before rifaximin treatment.; M, months. (**b**) Alb, albumin; The serum Alb levels significantly increased after RFX treatment from 3.2 (2.9–3.5) g/dL at baseline to 3.3 (2.9–3.6) g/dL, 3.3 (3.0–3.6) g/dL, 3.3 (3.0–3.6) g/dL, 3.4 (3.0–3.8) g/dL, 3.4 (3.1–3.7) g/dL, 3.4 (3.1–3.8) g/dL, 3.5 (3.1–3.8) g/dL after 3,6, 12, 18, 24, 30, and 36 months, respectively. A statistically significant difference was observed between baseline and after 3 months and more. Pre, before rifaximin treatment.; M, months. (**c**) PT (%), prothrombin time (%); PT activity significantly increased after RFX treatment from 69.0 (57.0–80.1) % at baseline to 69.5 (59.0–84.3) %, 71.0 (59.4–84.2) %, 72.0 (60.0–83.7) %, 73.0 (62.2–84.0) %, 72.8 (62.0–84.0) %, 79.0 (64.6–88.7) %, 80.6 (66.1–89.1) % after 3,6, 12, 18, 24, 30, and 36 months, respectively. A statistically significant difference was observed between baseline and after 3 months and more. Pre, before rifaximin treatment.; M, months.

Ammonia levels significantly decreased after 3 months of RFX administration and remained reduced over 3 years in all patients (*p* < 0.001; Figure 2a). This reduction was observed in both males and females (*p* < 0.001 and *p* < 0.05; Figure S1a,b), in elderly patients (≥65 years) and nonelderly patients (<65 years; *p* < 0.001 and *p* < 0.001; Figure S1c,d), and in patients with covert hepatic encephalopathy (CHE) and overt hepatic encephalopathy (OHE; *p* < 0.001 for both; Figure S1e,f).

Serum ALB levels significantly increased after 3 years of RFX treatment (*p* < 0.001; Figure 2b). This increase was observed in females but not in males (*p* = 0.0011 and *p* = 0.08; Figure S2a,b), in elderly patients (≥65 years) but not in nonelderly patients (<65 years; *p* = 0.002 and *p* = 0.23; Figure S2c,d), and in patients with CHE but not in those with OHE (*p* = 0.0022 and *p* = 0.16; Figure S2e,f).

The PT (%) activity increased significantly after 3 years of RFX treatment (*p* < 0.001; Figure 2c). This increase was observed in females but not in males (*p* = 0.021 and *p* = 0.029; Figure S3a,b), in nonelderly patients (<65 years) but not in elderly patients (≥65 years; *p* = 0.074 and *p* = 0.002; Figure S3c,d), and in patients with both CHE and OHE (*p* = 0.016 and *p* = 0.0043; Figure S2e,f).

No significant differences in serum total bilirubin alanine aminotransferase, aspartate aminotransferase, or total protein levels were observed.

### 3.3. The Cumulative Occurrence of OHE and Survival Rate

**Figure 3:**
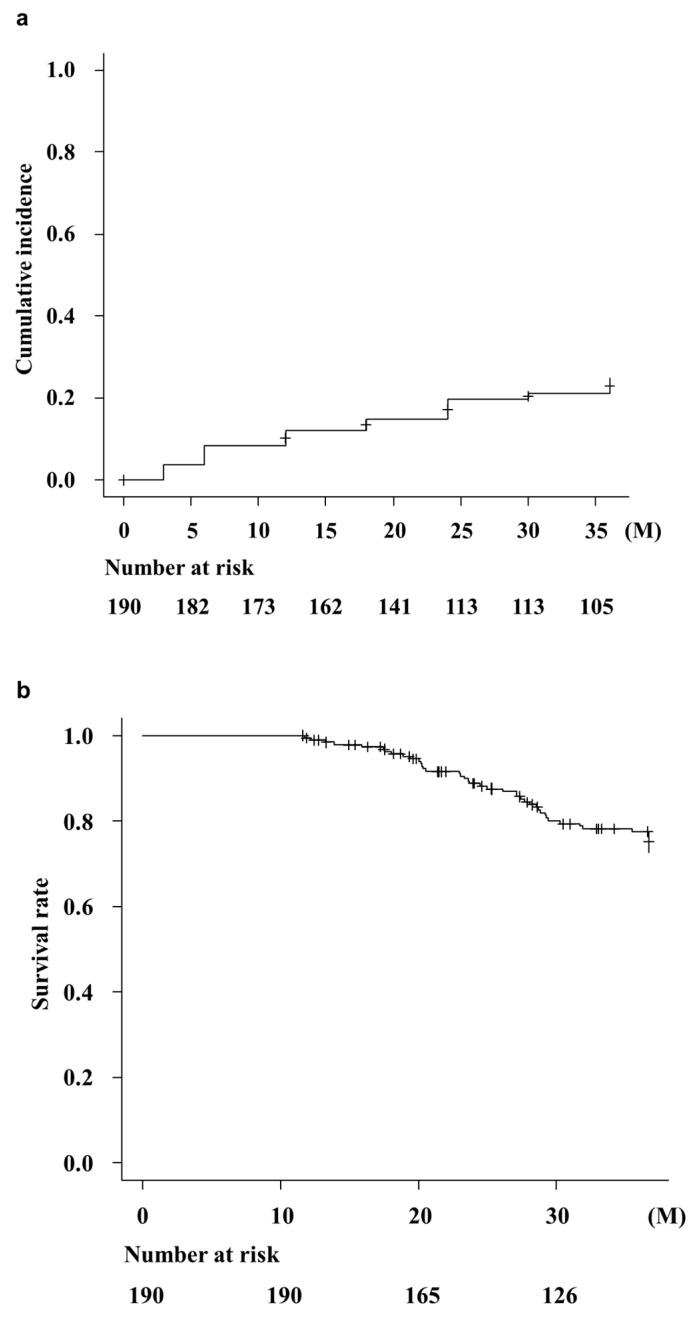
Cumulative incidence of OHE and survival rate of patients after RFX administration. (**a**) The rate of overt HE was 12.1%, 19.7%, and 24.9% at 1, 2, and 3 years, respectively. M, months. (**b**) Survival rate of the patients after RFX treatment. Survival rate after 1 year of RFX treatment was 100% and the survival rates after 2 and 3 years of RFX treatment were 88.9% (95%CI: 0.830–0.926) and 77.8% (95%CI: 0.703–0.831), respectively. M, months.

The cumulative incidence of OHE was 12.1%, 19.7%, and 24.9% within 1, 2, and 3 years, respectively. (Figure 3a). Median survival was not reached after 3 years of RFX administration. The survival rate at 1 year of treatment was 100% and survival rates at 2 and 3 years of RFX treatment were 88.9% (95%CI: 0.830–0.926) and 77.8% (95%CI: 0.703–0.831), respectively (Figure 3b). Of the 190 patients, the cause of death was recorded for 43 those who died. Of these, death was liver-related for 89% [*n* = 38; liver decompensation (*n* = 24, 56%), liver cancer (*n* = 13, 31%), and infection (*n* = 1, 2%)].

### 3.4. Safety of RFX Treatment

AST and ALT were unaffected during RFX treatment. BUN and Cr were also not affected during RFX treatment. Serum Na and K levels did not vary during RFX treatment. No patient experienced any serious adverse events (AEs) associated with renal function. A total of 15 (7.9%) patients had diarrhea. Twelve patients experienced improvement by a decrease in RFX dose or the administration of probiotics. Only three (1.6%) patients discontinued RFX therapy because of severe diarrhea; however, HE did not develop in these patients even after RFX discontinuation. Forty-three patients died in the follow-up period. No serious AEs, including liver failure, bleeding from esophagogastric varices, or spontaneous bacterial peritonitis, developed during the 3-year study period. No other AE were observed during the study period. Twenty-three patients stopped RFX after a year because of a follow-up interruption for hospital transfer. Eight patients stopped RFX after 1 year of RFX initiation because of improvement in HE. The patients (*n* = 113) were continuously given RFX for 3 years. Laboratory data from patients who died of liver disease within the first year of RFX initiation (*n* = 43) were excluded from the analysis.

## 4. Discussion

This multicenter 3-year follow-up study provided evidence for the long-term safety and efficacy of RFX treatment. We previously reported on the safety and efficacy of 12 months of RFX treatment in this cohort [24]. Three years of RFX treatment resulted in a continuous decrease in ammonia levels, whereas a continuous increase in Alb and PT activity was observed. Moreover, renal function (BUN and Cr) and electrolytes (Na and K) did not change after 3 years of RFX treatment. Furthermore, only a few occurrences of OHE were reported (The cumulative occurrence of OHE was 24.9% at 3 years). This suggests that RFX treatment has good efficacy and safety over 3 years of RFX treatment and may be used without discontinuation.

The results of meta-analysis of 28 randomized controlled trials show the efficacy and safety of RFX in patients with HE, indicating that RFX significantly improved HE grade, cognitive impairment, and prevented recurrent episodes [25]. A recent systematic review and network meta-analysis revealed that lactulose plus RFX significantly improved the incidence of OHE compared with the placebo group (RR = 0.19, 95% CI [0.09; 0.40]) [26]. This suggests that RFX strongly improves HE. To assess HE, WHC and ISHEN have been used [27]; however, both are inadequate for identifying CHE. Ammonia levels alone are not associated with neurologic complications or the severity of HE [3]; however, when using ammonia-lowering medicine used for the management of high blood levels of ammonia, repeated ammonia measurements are helpful for the treatment response. Neuropsychological assessment is useful for detecting CHE^,^ [28]. Herein, these tests were not performed; rather, the diagnosis of CHE was determined based on ammonia levels or the attending physician’s assessment of the clinical symptoms.

RFX was first approved for HE in Italy in 1985 [29]; however, it was not approved in Japan until November 2016. RFX prevents the incidence and recurrence of HE and lowers HE incidence during stay in hospital [30]. The safety and effectiveness of long-term RFX treatment have already been reported in Europe and the USA [17]. By contrast, in Japan, the safety and effectiveness of RFX have only been evaluated for up to 12 weeks of treatment [15]; thus, little is known regarding the efficacy of long-term treatment with RFX [16,17,18,19,20,21]. First-line treatment for HE includes synthesized disaccharides, including lactulose. RFX is used for HE as second-line treatment. The most frequent AEs with lactulose were distaste, diarrhea, and abdominal bloating; however, the side effect of lactulose is dose-dependent [31]. AEs with RFX comprise diarrhea and constipation, and severe Clostridium difficile infection (CDI). The CDI rate remained stable with long-term RFX treatment [20]. The percentages of the AEs are significantly higher for lactulose treatment than for RFX treatment. A single center, retrospective study revealed the long-term tolerability of RFX versus lactulose [32]. L-ornithine-L-aspartate infusions were found to be effective in patients with cirrhosis and HE [30]. Meta-analyses of randomized trials show that lactulose reduces mortality in patients with HE [31]. L-Carnitine addition decreased the risk of hospitalization in patients with HE treated with rifaximin [33]. Several studies have shown that combination of lactulose plus rifaximin is more effective than lactulose alone in the treatment of OHE [5,34,35,36]. A randomized, double-blind controlled trial demonstrated that the combination of RFX plus lactulose is effective for HE recurrence [37]. Furthermore, there are add-on effects of levocarnitine and Zinc to RFX [26]. A phase III clinical trial of RFX showed that AEs developed in 13.4% of the patients including 6.4% of the patients developing gastrointestinal AEs at 12 weeks [15]. With respect to AEs in our previous study, 15 (7.9%) patients had diarrhea. RFX dose reduction or probiotic supplementation improved diarrhea in 12 patients. Only three (1.6%) patients discontinued RFX treatment owing to severe diarrhea; however, HE did not develop in these patients even after the withdrawal of RFX. The patients who withdrew RFX before 1-year RFX continuation were excluded for different reasons including AEs. The incidence of AEs owing to RFX treatment remained unclear; however, no other serious adverse events were observed following RFX administration. Prospective studies are required to determine the efficacy and safety of RFX on patients with HE.

In the present study, liver function reserve improved 3 years after RFX treatment. Previous reports indicated that treatment with a nucleoside analog for hepatitis B [38], direct-acting antivirals for hepatitis C [39], or balloon occulted retrograde transfemoral obliteration for gastric varices [40] improved liver function reserve. The results showed that the long-term administration of RFX for 3 years improves the liver function reserve. The improvement in liver function reserve by RFX treatment remains unclear but could be partially accounted for by the fact that RFX treatment improves nutritional status assessed by a controlling nutritional status score [16]. The long-term RFX improves dietary intake resulting from HE improvement. Second, RFX improves small intestinal bacterial overgrowth caused by improvement of suppressed intestinal motility and delayed bowel transit time [41], resulting in amelioration of malabsorption in patients with HE [42]. RFX also improved cognition and reduced endotoxin activity without affecting the composition of the gut microbiome [43] by ameliorating the gut barrier function and bacterial translocation in patients with cirrhosis [44].

Once OHE occurs, patient prognosis worsens, and the survival rate within 1 year is 36% and 15% within 5 years [45]. The survival rate of patients with HE not receiving RFX treatment was 44% [42]. One-year survival in the HE cohorts who were administered RFX was 48.3% [43]. Notably, RFX reduced the risk of mortality in patients with HE in a multivariable Cox model of survival [44]. As this study has a bias for patients who had been on continuous administration for 1 year were eligible, the exact prognosis at 1 and 2 years was 88.9% and 77.8%, respectively. Nevertheless, the survival data were superior to the previous report. The results of the present study indicate that long-term RFX treatment improves patient prognosis.

This study had several limitations. First, this study employed a retrospective design, potentially lacking the adequate power to determine the efficacy of rifaximin. Second, we did not perform the neuropsychological tests to diagnose CHE and the improvement of CHE could not be proven objectively. Third, the criteria for RFX administration differs with the medical facilities. Third, medications already prescribed to treat hyperammonemia, including disaccharide lactulose, BCAA supplements, levocarnitine, and zinc, were not discontinued during RFX administration. Herein, the number of patients who administered disaccharide lactulose, and BCAA were 62 patients and 21 patients, respectively. However, the number of patients who were prescribed levocarnitine and zinc was unclear because of the multicenter nature of this study. Fourth, selection bias may have arisen because the patients administered RFX for over 1 year were enrolled. Fifth, this study did not investigate the effects of rifaximin on gut microbiota. Sixth, the associations with improved outcomes could be attributed to various factors, including the treatment of underlying liver disease, the overall timeframe of improved clinical care, and modifications of risk factors such as alcohol use and hepatitis C elimination. Seventh, as this study is single-arm in design, the possibility of spontaneous disease progression over time, even without drug administration, cannot be ruled out. Generally, chronic liver disease is a condition that progressively worsens, making theories such as regression to the mean inapplicable. If no changes in the laboratory data are observed, RFX may still be considered effective. However, it remains challenging to determine whether the observed improvements in laboratory values were directly due to rifaximin or to other medications and factors, as the study lacked sufficient data on concurrent treatments and contributing factors. Eighth, there was a difference in efficacy of RFX between the groups stratified based on gender, age, and severity of HE.

Therefore, future studies should prospectively evaluate the effects of RFX on patients with HE.

In summary, this multicenter retrospective study suggests that rifaximin treatment over 3 years is both effective and safe for patients with HE. Significant improvements in ammonia levels, serum albumin, and prothrombin activity indicate that rifaximin positively impacts liver function, which is crucial for managing HE. The relatively low incidence of overt HE (12.1%, 19.7%, and 24.9% at 1, 2, and 3 years, respectively) and high survival rates (100%, 88.9%, and 77.8% at 1, 2, and 3 years) further support the long-term efficacy of rifaximin. The treatment was well tolerated, with only a small proportion of patients discontinuing due to adverse effects, primarily severe diarrhea. No significant changes were observed in BUN, Cre, Na, or K, whereas Alb and PT activity significantly increased after 3 years of RFX treatment compared with pretreatment levels. These findings suggest that long-term RFX administration does not adversely affect kidney function or electrolyte balance. Importantly, no significant changes in renal function or electrolyte levels were observed, indicating that rifaximin does not negatively impact these parameters. Overall, this study highlights the significant benefits of rifaximin for patients with HE.

## 5. Conclusions

We concluded that 3 years of continuous rifaximin (RFX) treatment was both effective and safe for patients with hepatic encephalopathy. Liver function did not worsen during treatment. Notably, this study is the first multicenter, 3-year follow-up investigation providing evidence for the long-term safety and efficacy of RFX in Japanese patients with hepatic encephalopathy.

## Figures and Tables

**Table 1:** Clinical characteristics of patients with cirrhosis.

**Variables**	**(*n* = 190)**
Age (years)	71.0 (64.0–77.0)
Sex (male/female)	116/74
Body mass index (kg/m^2^)	24.4 ± 4.6
Etiology (HBV/HCV/alcohol/MASLD/other)	19/33/57/27/54
Child–Pugh grade (A/B/C)	14/154/22
History of HE (yes/no)	47/143
Type of HE (overt/covert)	30/160
Esophagogastric varices (yes/no)	105/85
Portosystemic shunt (yes/no)	99/91
Splenomegaly (yes/no)	143/47
Ascites (yes/no)	69/121
Spontaneous bacterial peritonitis (yes/no)	0/190
Hepatocellular carcinoma (yes/no)	61/129
Stage of hepatocellular carcinoma (1/2/3/4)	21/27/8/5
Survival (alive/dead)	147/43
Concomitant medications (lactulose/BCAA)	62/21

BCAA, branched-chain amino acid; HBV, hepatitis B; HCV, hepatitis C; HE, hepatic encephalopathy; MASLD, metabolic dysfunction–associated steatotic liver disease.

**Table 2:** Clinical characteristics of patients with cirrhosis stratified by sex.

Variables	Male (*n* = 116)	Female (*n* = 74)	*p-*Value
Age (years), median (IQR)	68.5 (61.8–77.0)	73.5 (65.3–78.0)	0.049
Body mass index (kg/m^2^), median (IQR)	24.7 (22.1–28.7)	23.8 (20.7–26.4)	0.022
Etiology (HBV/HCV/alcohol/MASLD/other)	13/15/47/15/26	6/18/10/12/28	0.21
Child–Pugh (A/B/C)	7/92/17	7/62/5	0.19
History of OHE (yes/no)	23/93	24/50	0.059
Type of HE (overt/covert)	14/102	16/58	0.10
Esophagogastric varices (yes/no)	66/50	39/35	0.65
Portosystemic shunt (yes/no)	49/67	50/24	<0.001
Splenomegaly (yes/no)	89/27	54/20	0.61
Ascites (yes/no)	48/68	21/53	0.089
Spontaneous bacterial peritonitis (yes/no)	0/116	0/74	-
Hepatocellular carcinoma (yes/no)	44/72	17/57	0.038
Stage of hepatocellular carcinoma (1/2/3/4)	17/17/6/4	4/10/2/1	0.56
Survival (alive/dead)	91/25	56/18	0.72
Concomitant medications (lactulose/BCAA)	32/12	30/9	0.66

BCAA, branched-chain amino acid; HBV, hepatitis B; HCV, hepatitis C; HE, hepatic encephalopathy; IQR, interquartile range; OHE, overt hepatic encephalopathy; MASLD, metabolic dysfunction–associated steatotic liver disease.

**Table 3:** Clinical characteristics of patients with cirrhosis stratified by age.

Variables	≥65 Years Old (*n* = 135)	<65 Years Old (*n* = 55)	*p-*Value
Age (years), median (IQR)	75.0 (70.0–79.0)	59.0 (51.0–62.0)	<0.001
Sex (male/female)	75/60	41/14	0.021
Body mass index (kg/m^2^), median (IQR)	24.0 (21.8–26.7)	25.4 (20.9–28.7)	0.34
Etiology (HBV/HCV/alcohol/MASLD/other)	13/28/28/19/47	6/5/29/8/7	0.15
Child–Pugh (A/B/C)	10/117/8	4/37/14	0.94
History of HE (yes/no)	38/97	9/46	0.098
Type of HE (overt/covert)	25/110	5/50	0.13
Esophagogastric varices (yes/no)	74/61	31/24	0.87
Portosystemic shunt (yes/no)	74/61	25/30	0.27
Splenomegaly (yes/no)	96/39	47/8	0.042
Ascites (yes/no)	47/88	22/33	0.51
Spontaneous bacterial peritonitis (yes/no)	0/135	0/55	-
Hepatocellular carcinoma (yes/no)	45/90	16/39	0.61
Stage of hepatocellular carcinoma (1/2/3/4)	13/22/6/4	8/5/2/1	0.50
Survival (alive/dead)	107/28	40/15	0.34
Concomitant medications (lactulose/BCAA)	23/6	39/15	0.49

BCAA, branched-chain amino acid; HBV, hepatitis B; HCV, hepatitis C; HE, hepatic encephalopathy; IQR, interquartile range; MASLD, metabolic dysfunction–associated steatotic liver disease.

**Table 4:** Clinical characteristics of patients with cirrhosis stratified by type of hepatic encephalopathy.

Variables	Overt HE (*n* = 30)	Covert HE (*n* = 160)	*p-*Value
Age (years), median (IQR)	74.5 (69.3–78.0)	70.0 (63.0–77.0)	<0.001
Sex (male/female)	14/16	102/58	0.10
Body mass index (kg/m^2^), median (IQR)	24.0 (21.8–26.7)	25.4 (20.9–28.7)	0.34
Etiology (HBV/HCV/alcohol/MASLD/other)	2/6/7/8/7	17/27/50/19/47	0.31
Child–Pugh (A/B/C)	1/26/3	13/128/19	0.81
History of HE (yes/no)	14/16	129/31	<0.001
Esophagogastric varices (yes/no)	11/19	74/86	0.42
Portosystemic shunt (yes/no)	11/19	80/80	0.23
Splenomegaly (yes/no)	8/22	39/121	0.82
Ascites (yes/no)	16/14	105/55	0.22
Spontaneous bacterial peritonitis (yes/no)	0/30	0/160	-
Hepatocellular carcinoma (yes/no)	25/5	104/56	0.056
Stage of hepatocellular carcinoma (1/2/3/4)	3/1/0/1	18/26/8/4	0.29
Survival (alive/dead)	5/25	38/122	0.48
Concomitant medications (lactulose/BCAA)	33/7	29/14	0.11

BCAA, branched-chain amino acid; HBV, hepatitis B; HCV, hepatitis C; HE, hepatic encephalopathy; IQR, interquartile range; MASLD, metabolic dysfunction–associated steatotic liver disease.

**Table 5:** Pretreatment laboratory values of patients with hepatic encephalopathy.

Variables	Median (IQR)
Hemoglobin (g/dL)	11.8 (10.3–13.4)
Platelets (1 × 10^4^/L)	10.0 (7.2–13.2)
Prothrombin activity (%)	67.9 (54.0–79.7)
Total protein (g/dL)	7.0 (6.4–7.4)
Serum albumin (g/dL)	3.2 (2.9–3.6)
Aspartate aminotransferase (U/L)	40.5 (29.8–53.3)
Alanine aminotransferase (U/L)	25.0 (18.0–37.0)
Total bilirubin (mg/dL)	1.4 (0.9–2.3)
Serum ammonia (g/dL)	99.0 (64.8–140.0)
Blood urine nitrogen (mg/dL)	15.0 (10.0–19.9)
Serum creatine (mg/dL)	0.8 (0.6–1.0)
Serum sodium (mEq/L)	139.0 (137.0–141.0)
Serum potassium (mEq/L)	4.2 (3.9–4.6)
ALBI score	−1.33 (−1.95–1.54)
MELD score	10.6 (8.4–12.3)

N = 190. ALBI, albumin–bilirubin; IQR, interquartile range; MELD, Model for End-Stage Liver Disease.

## Data Availability

Derived data supporting the findings of this study are available from the corresponding author [T.N.] on request.

## Supplementary Materials

The following supporting information can be downloaded at: https://www.mdpi.com/article/10.3390/jcm14041358/s1, Figure S1a: male NH3, Figure S1b: female NH3, Figure S1c: 65 years old ≦ NH3, Figure S1d: 65 years old > NH3, Figure S1e: overt hepatic encephalopathy NH3, Figure S1f: covert hepatic encephalopathy NH3; Figure S2a: male albumin, Figure S2b: female albumin, Figure S2c: 65 years old ≦ albumin, Figure S2d: NH3: 65 years old > albumin, Figure S2e: overt hepatic encephalopathy albumin, Figure S2f: covert hepatic encephalopathy albumin; Figure S3a: male pro thrombin (%), Figure S3b: female pro thrombin (%), Figure S3c: 65 years old ≦ pro thrombin (%), Figure S3d: NH3: 65 years old > pro thrombin (%), Figure S3e: overt hepatic encephalopathy pro thrombin (%), Figure S3f: covert hepatic encephalopathy pro thrombin (%).
