# Supplementary material for: Real-World Setting of Efficacy and Safety of 3 Years of Rifaximin Administration in Japanese Patients with Hepatic Encephalopathy: A Multicenter Retrospective Study
Source: J Clin Med. 2025 Feb 18;14(4):1358. doi: 10.3390/jcm14041358 (PMC11856843; doi:10.3390/jcm14041358)

Figure S1a

Male

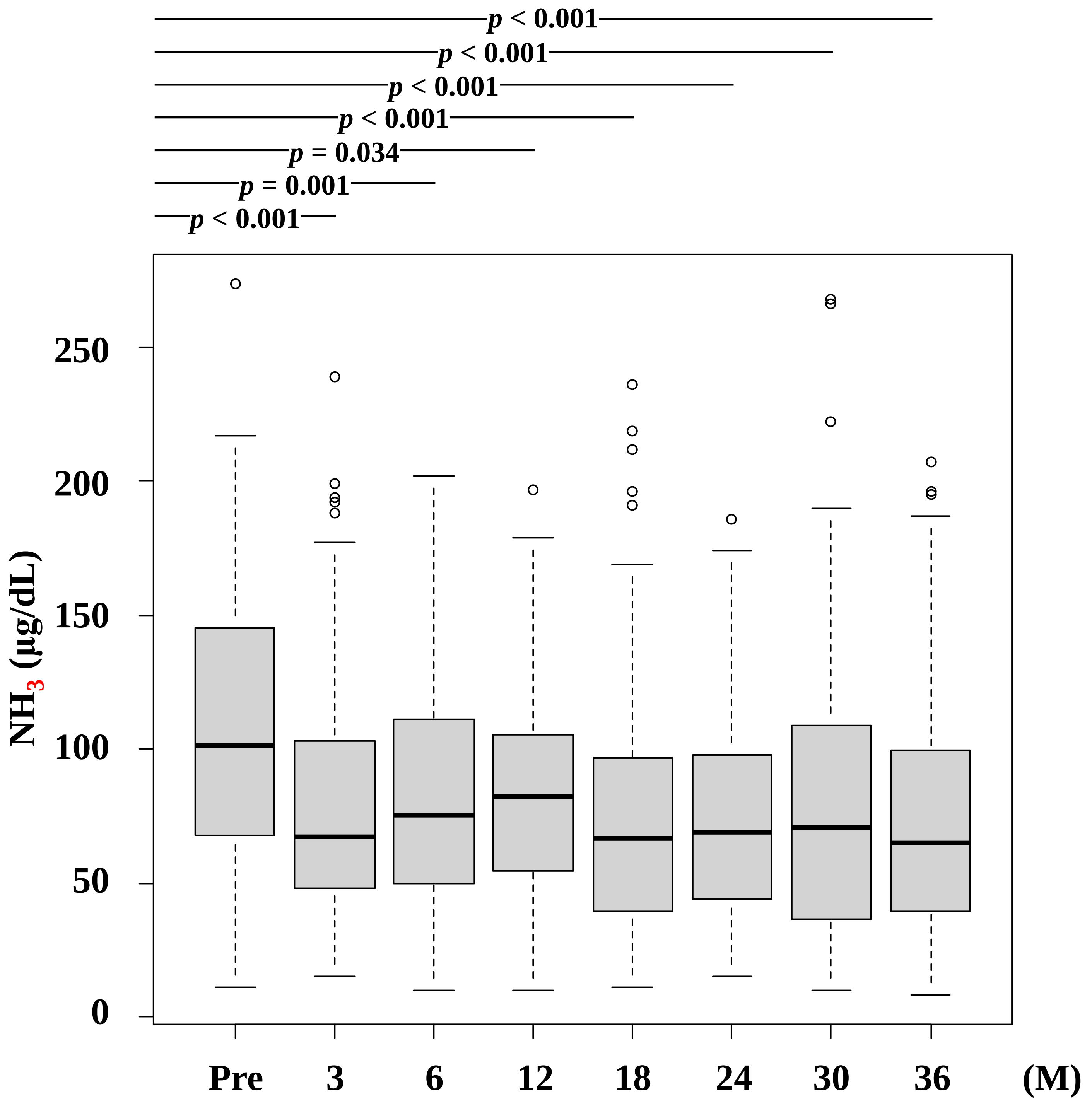

Figure S1b

Female

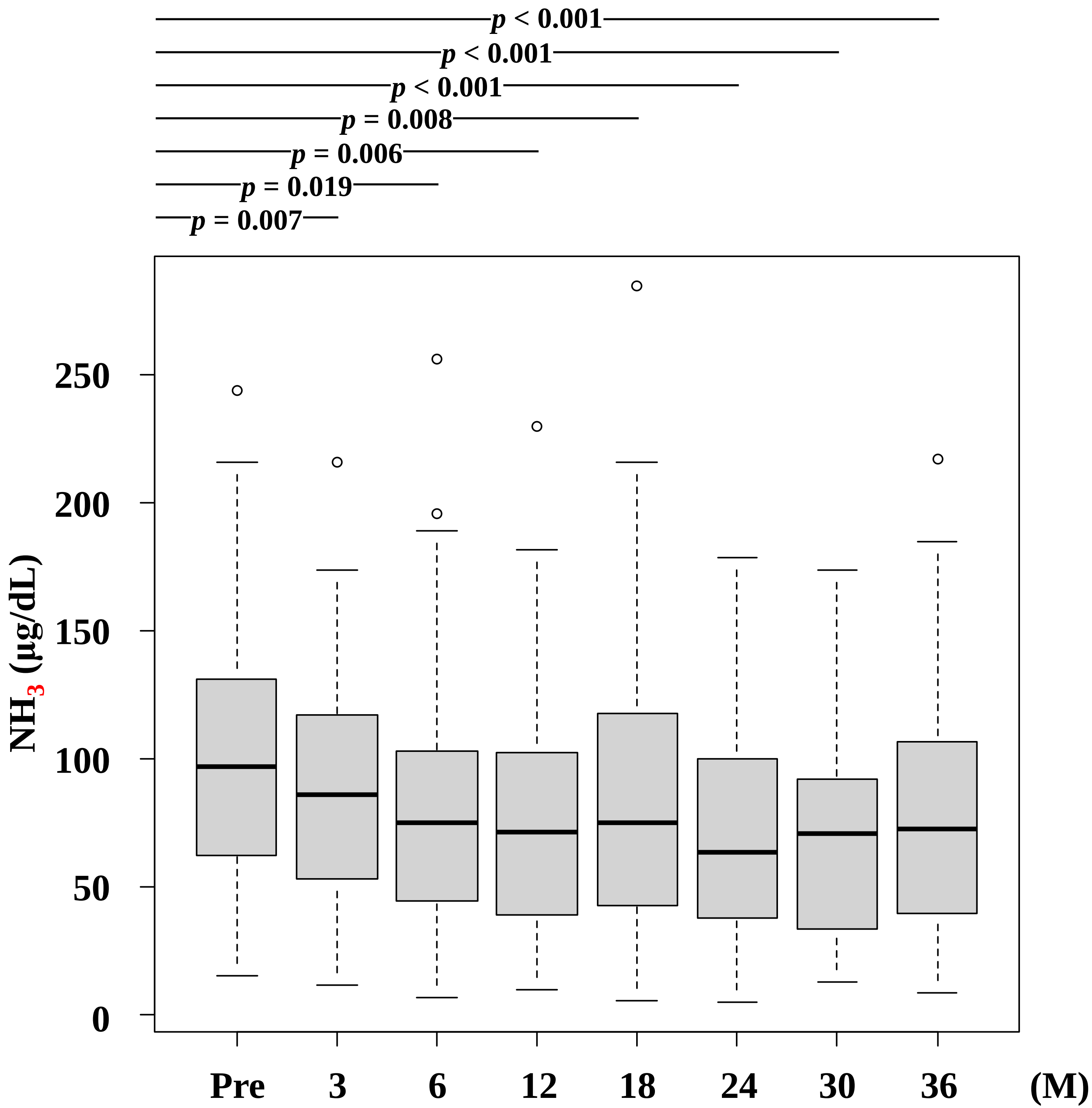

Figure S1c

＜65 years old

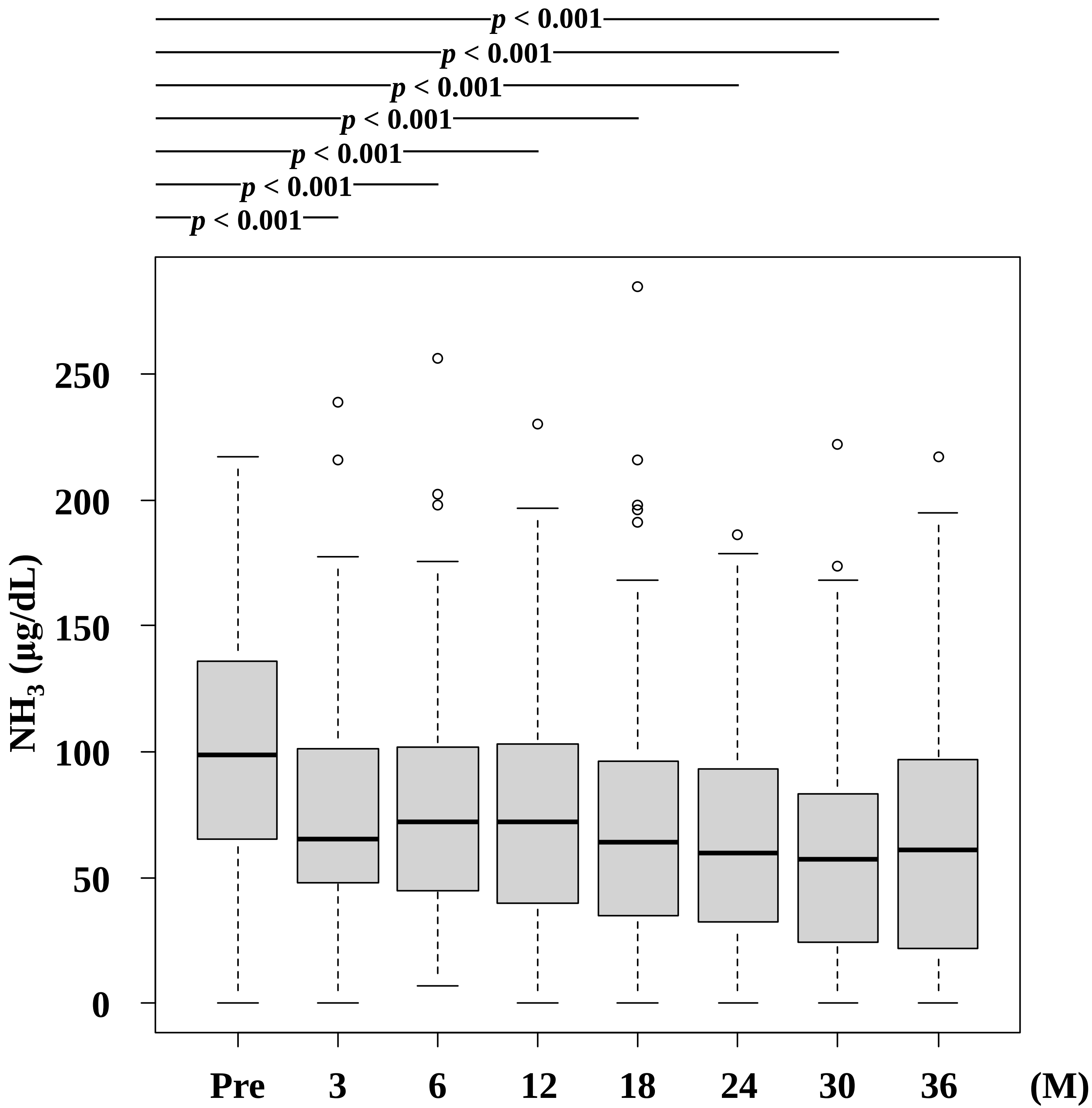

Figure S1d

≧65 years old

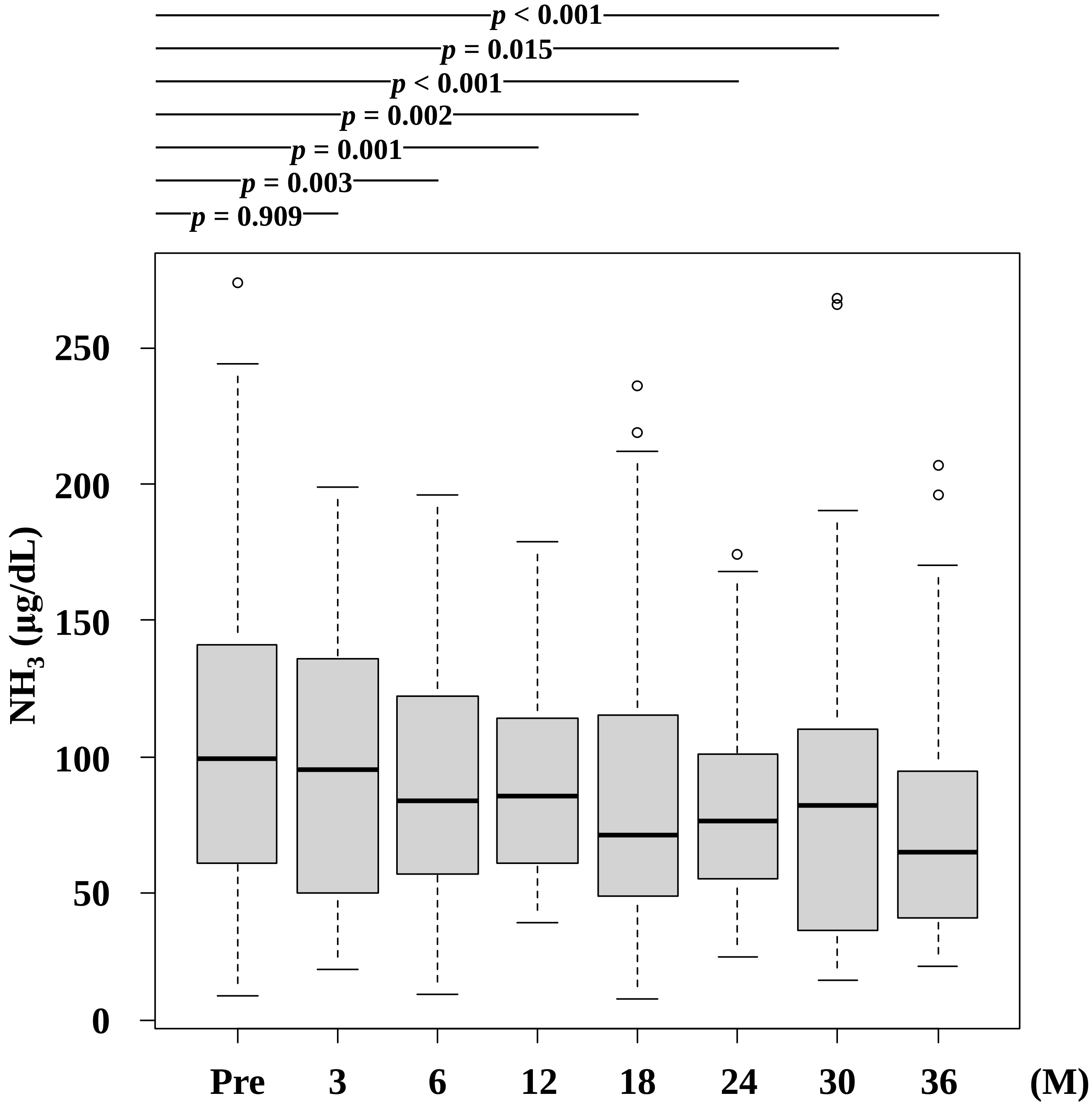

Figure S1e

Covert hepatic encephalopathy

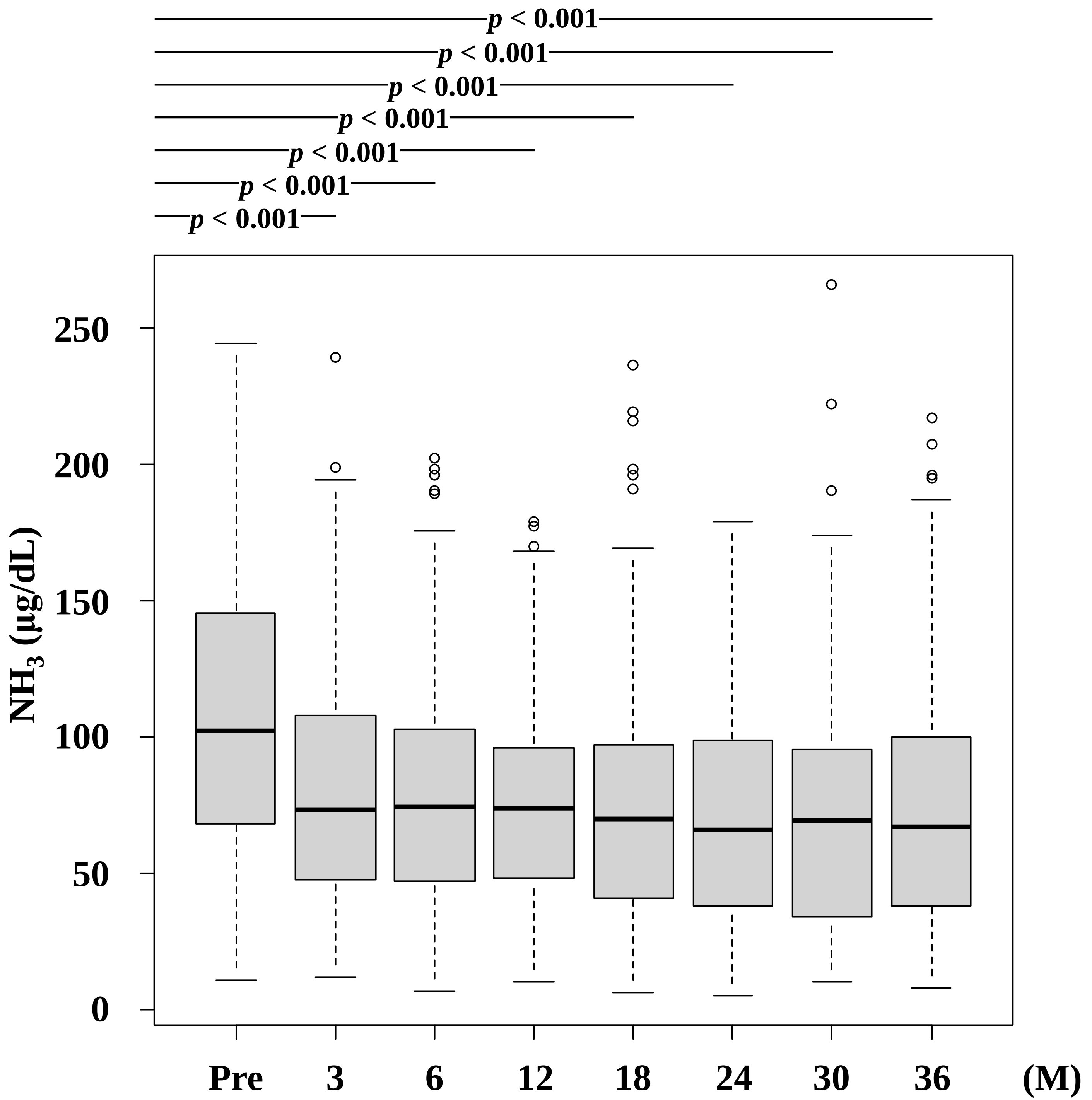

Figure S1f

Overt hepatic encephalopathy

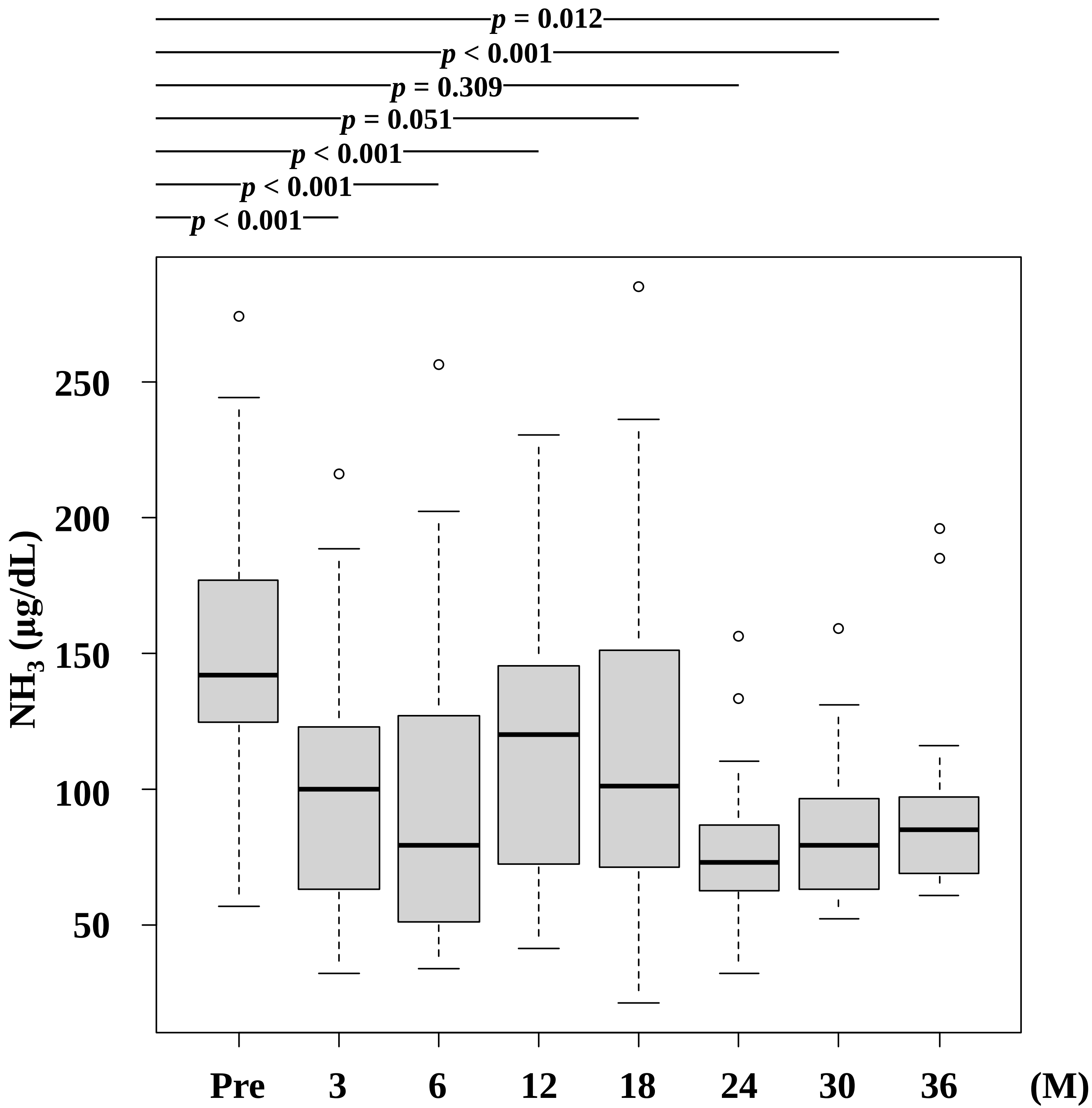

Figure S2a

Female

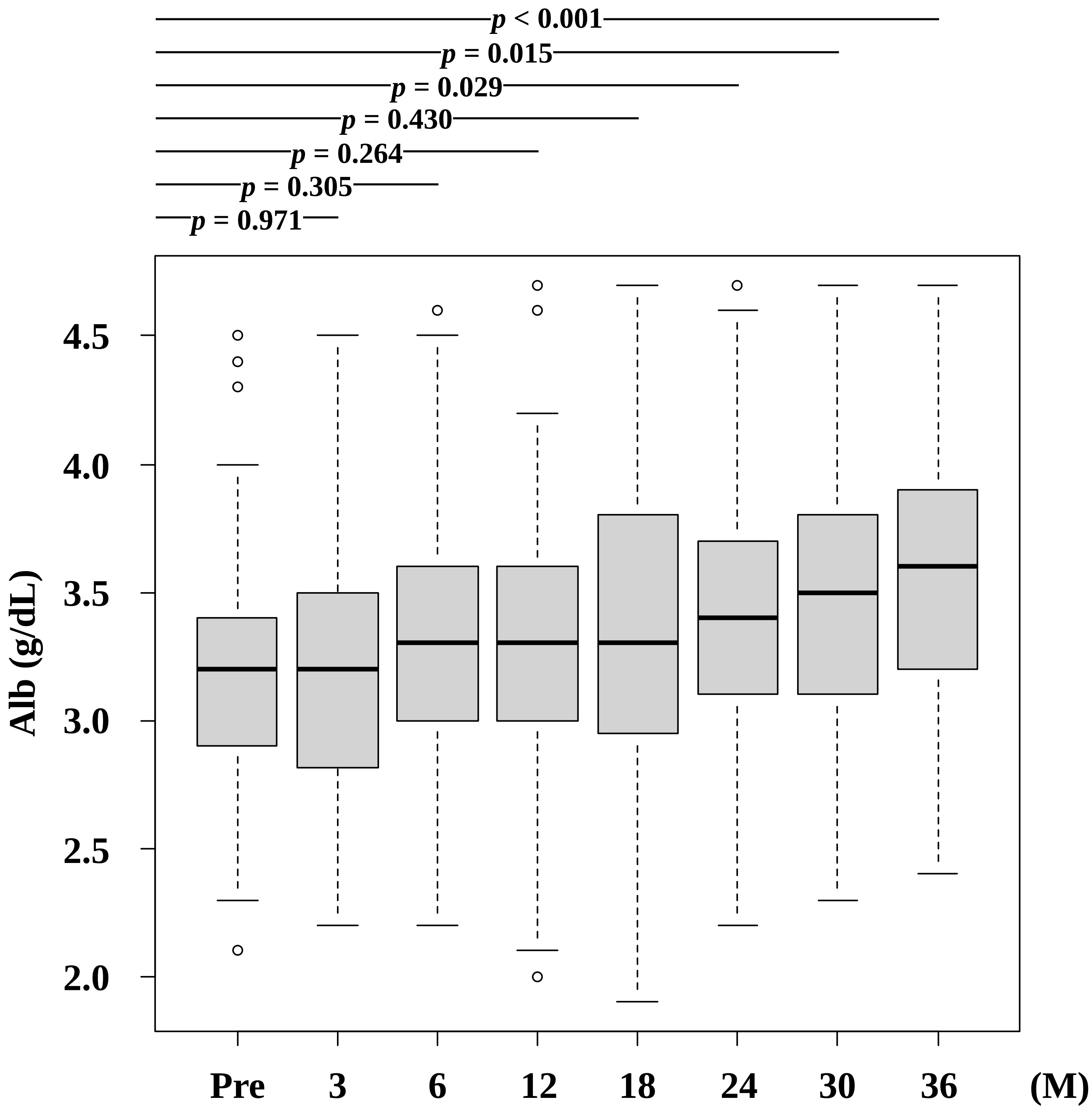

Figure S2b

Male

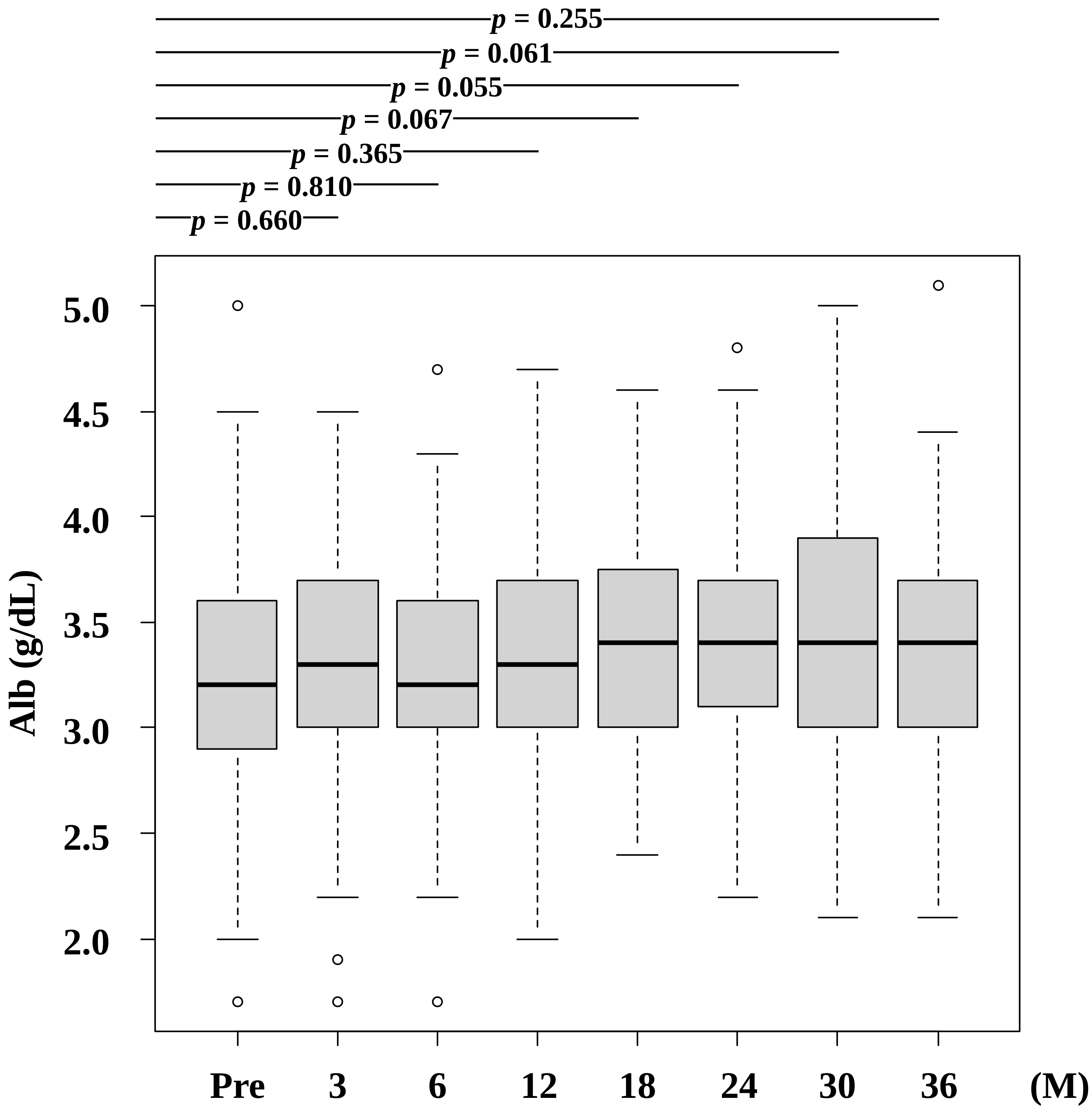

Figure S2c

＜65 years old

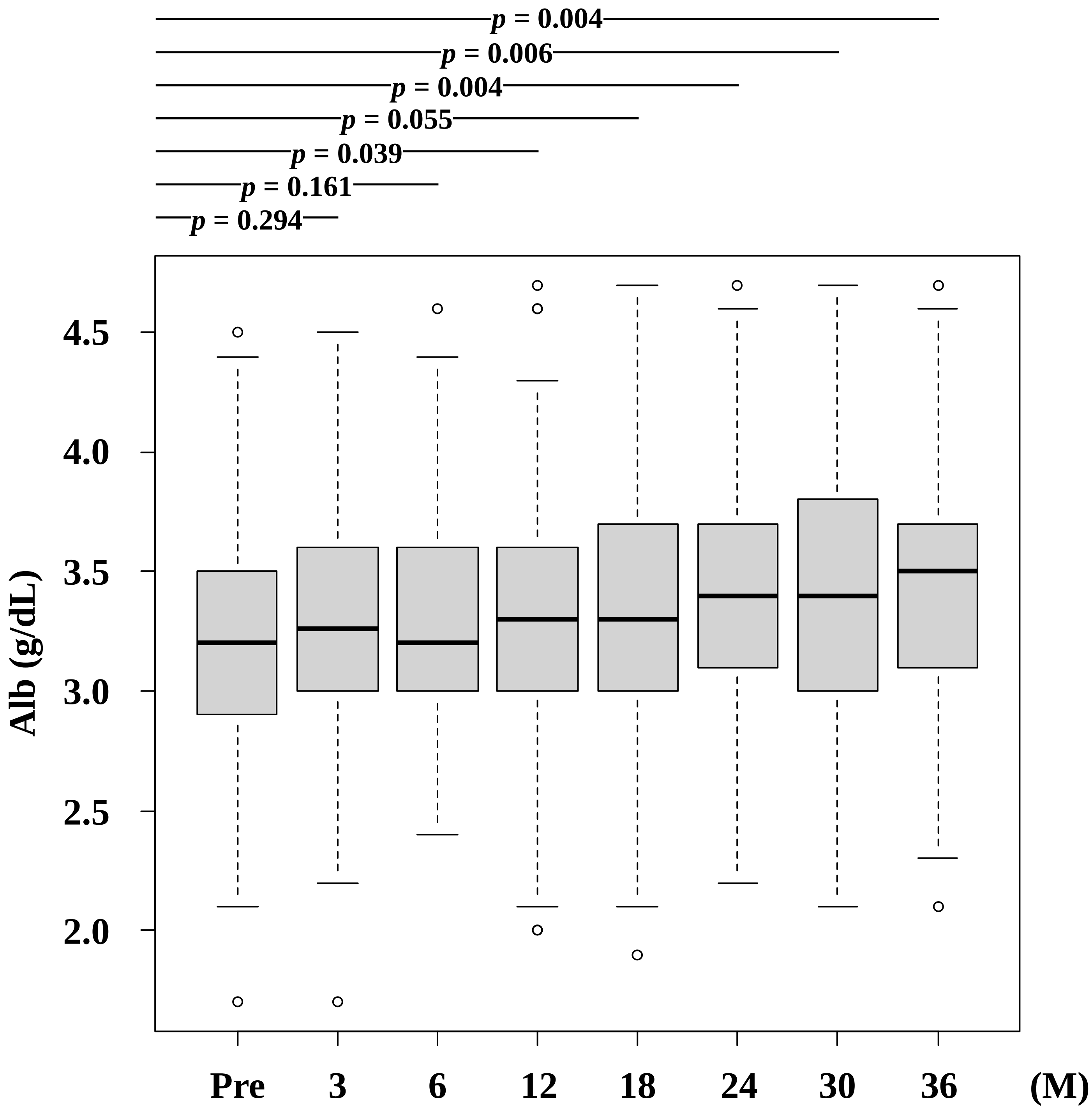

Figure S2d

≧65 years old

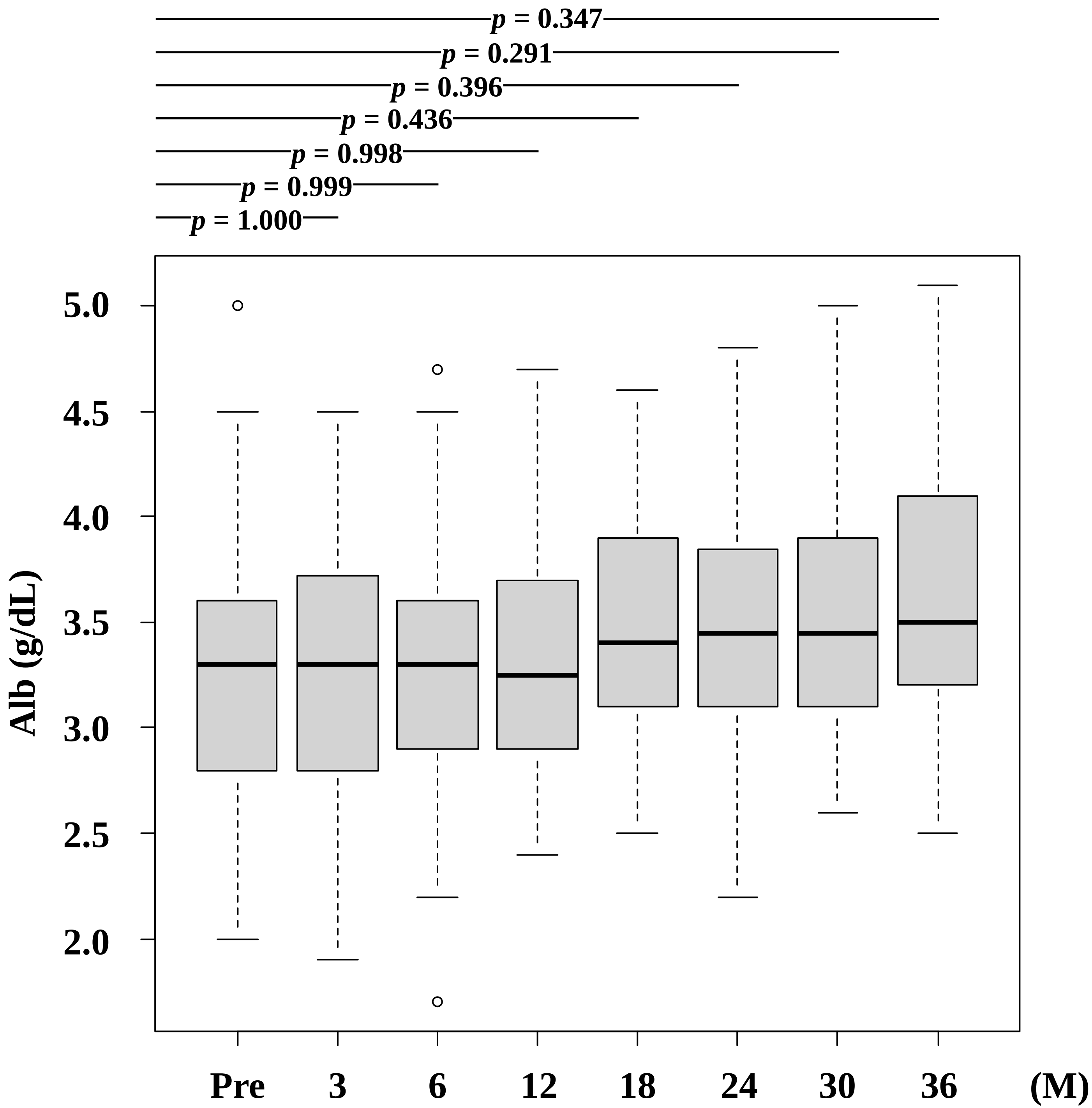

Figure S2e

Covert hepatic encephalopathy

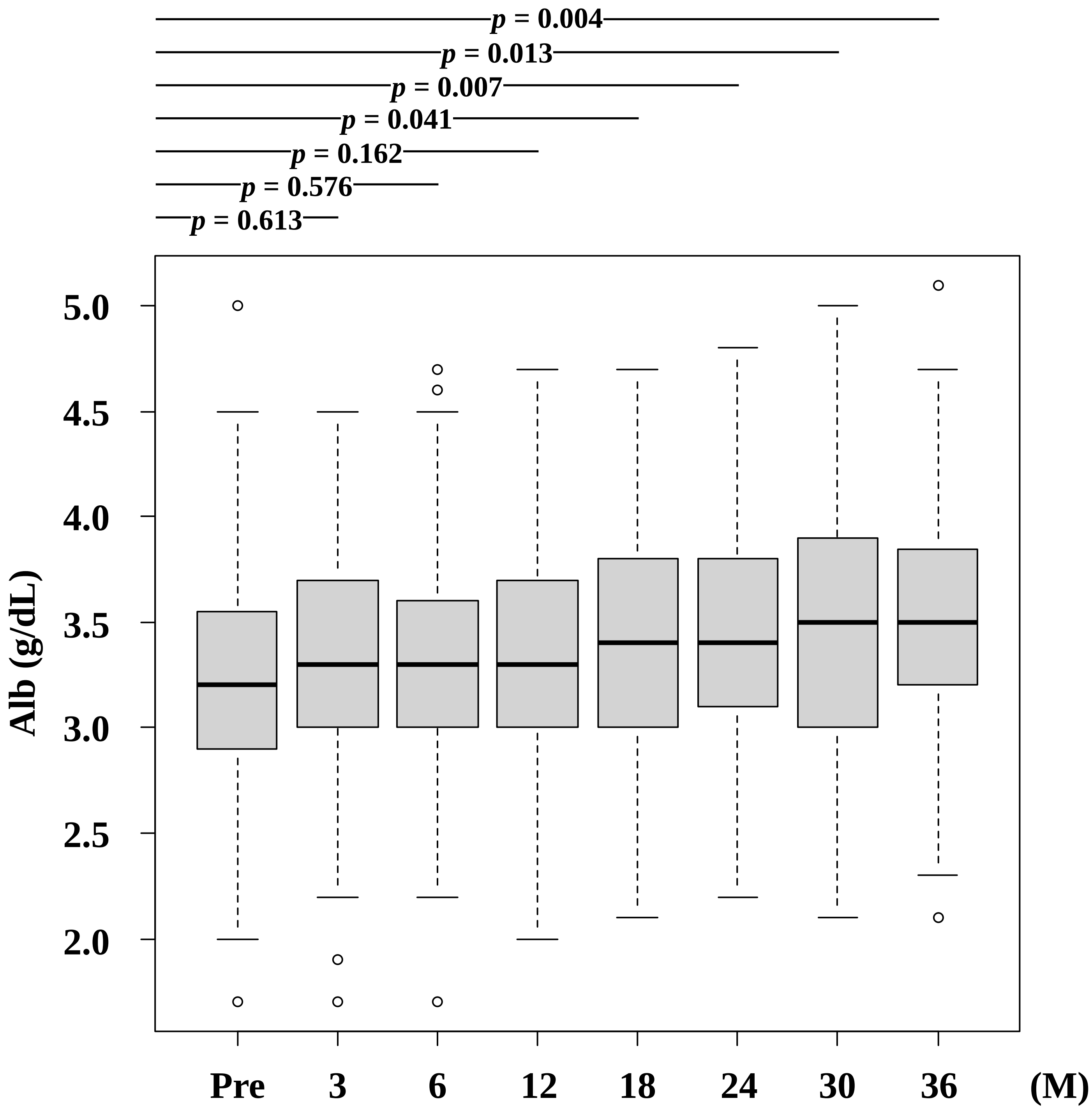

Figure S2f

Overt hepatic encephalopathy

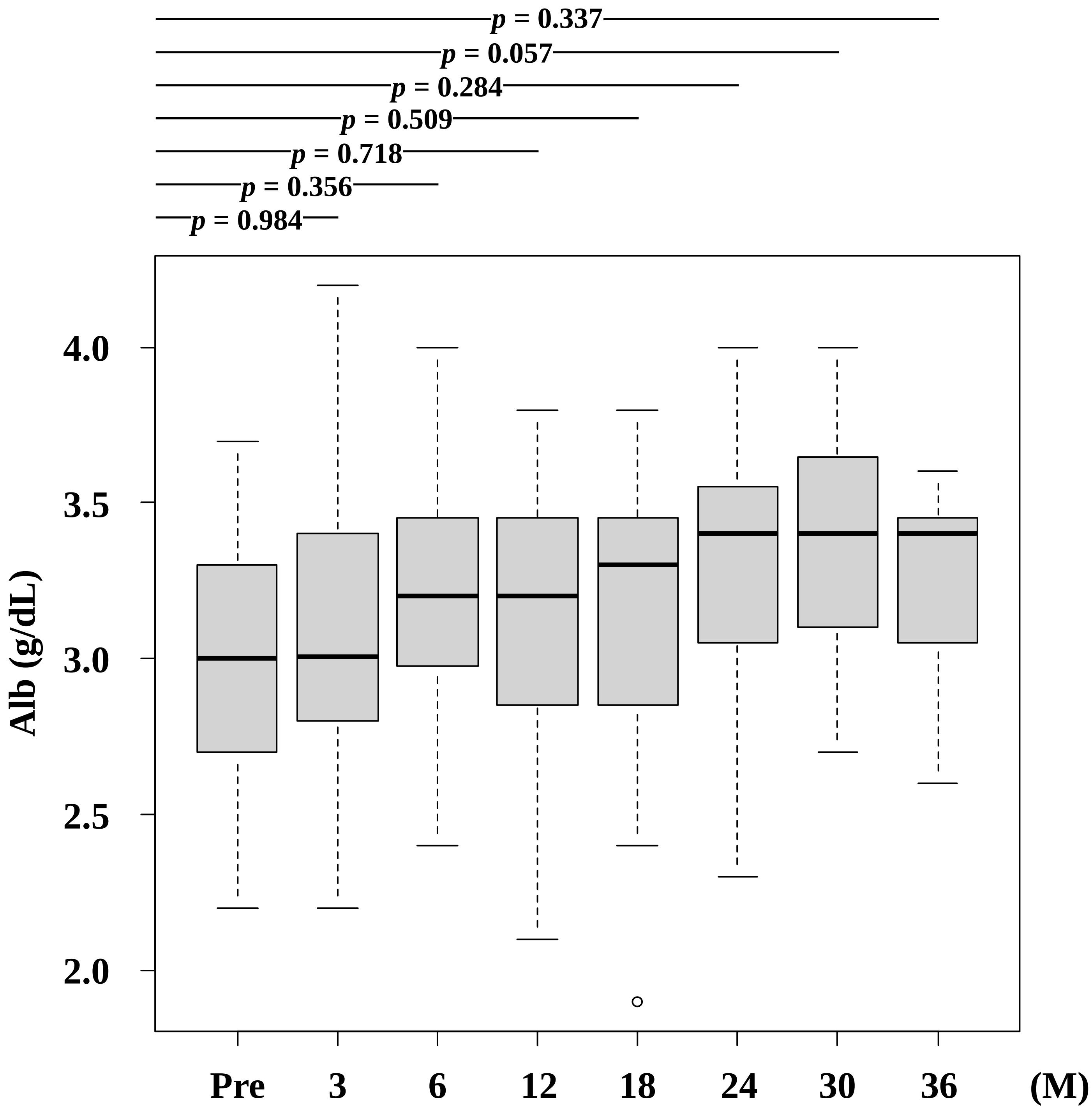

Figure S3a

Male

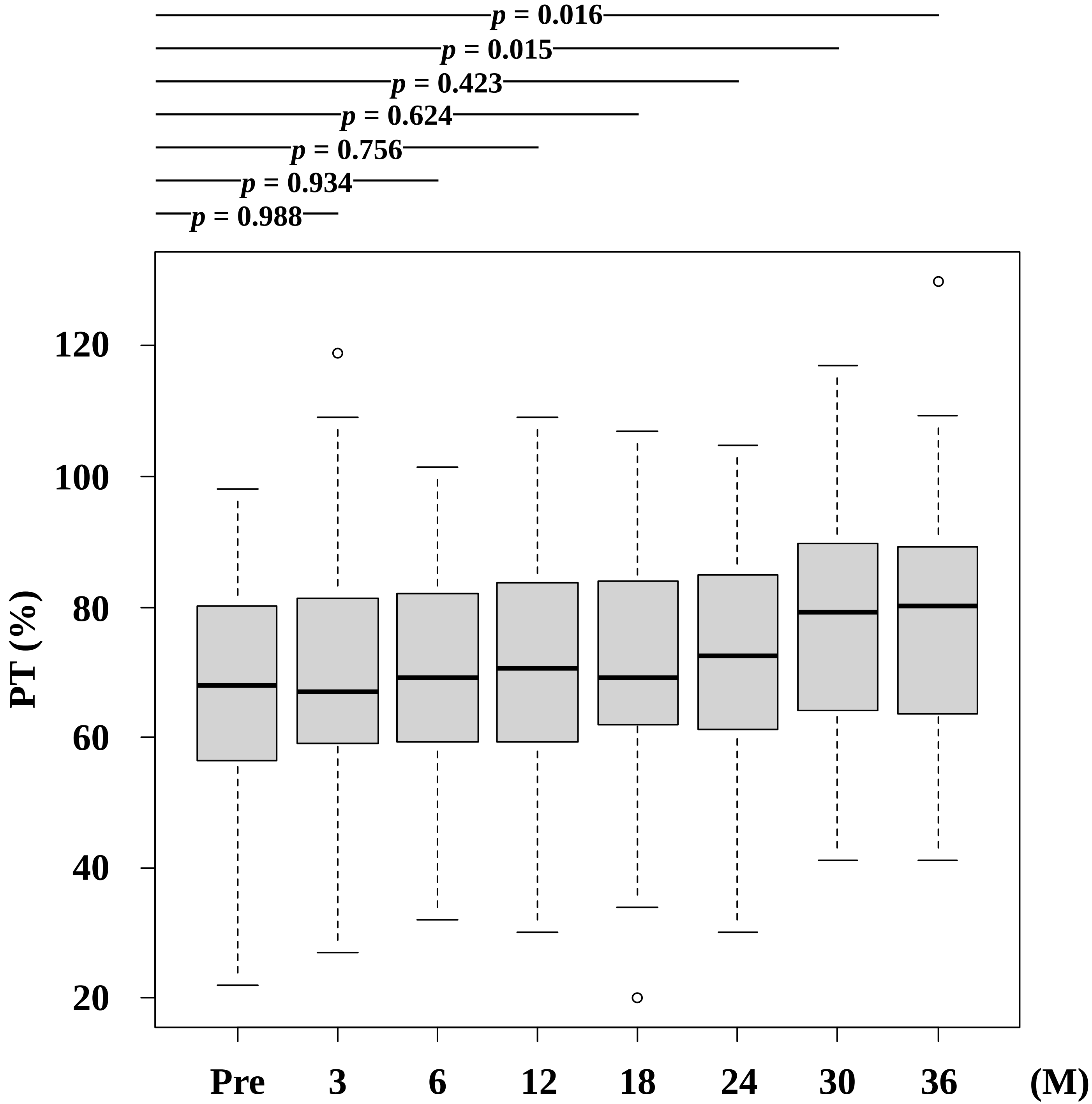

Figure S3b

Female

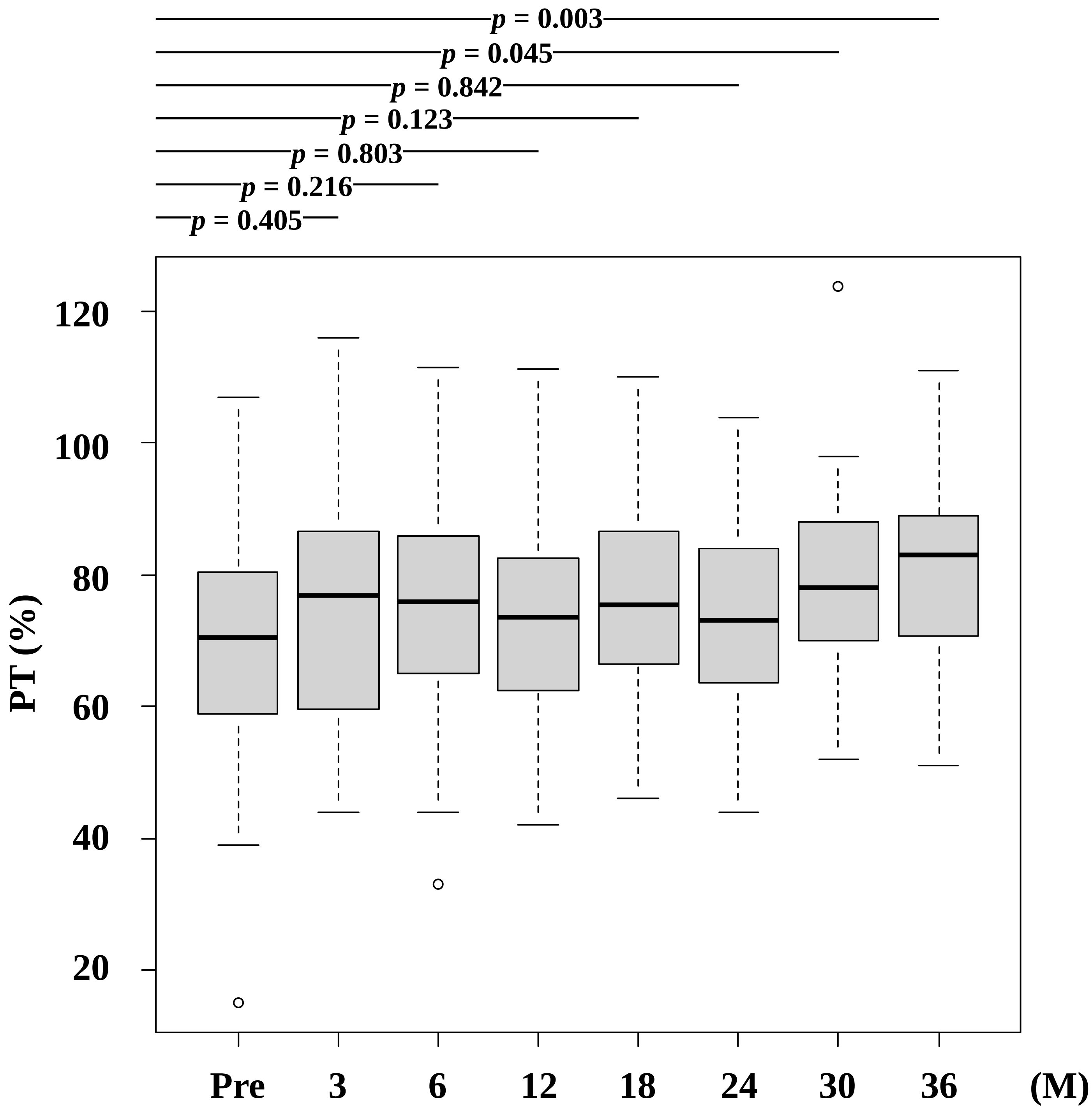

Figure S3c

＜65 years old

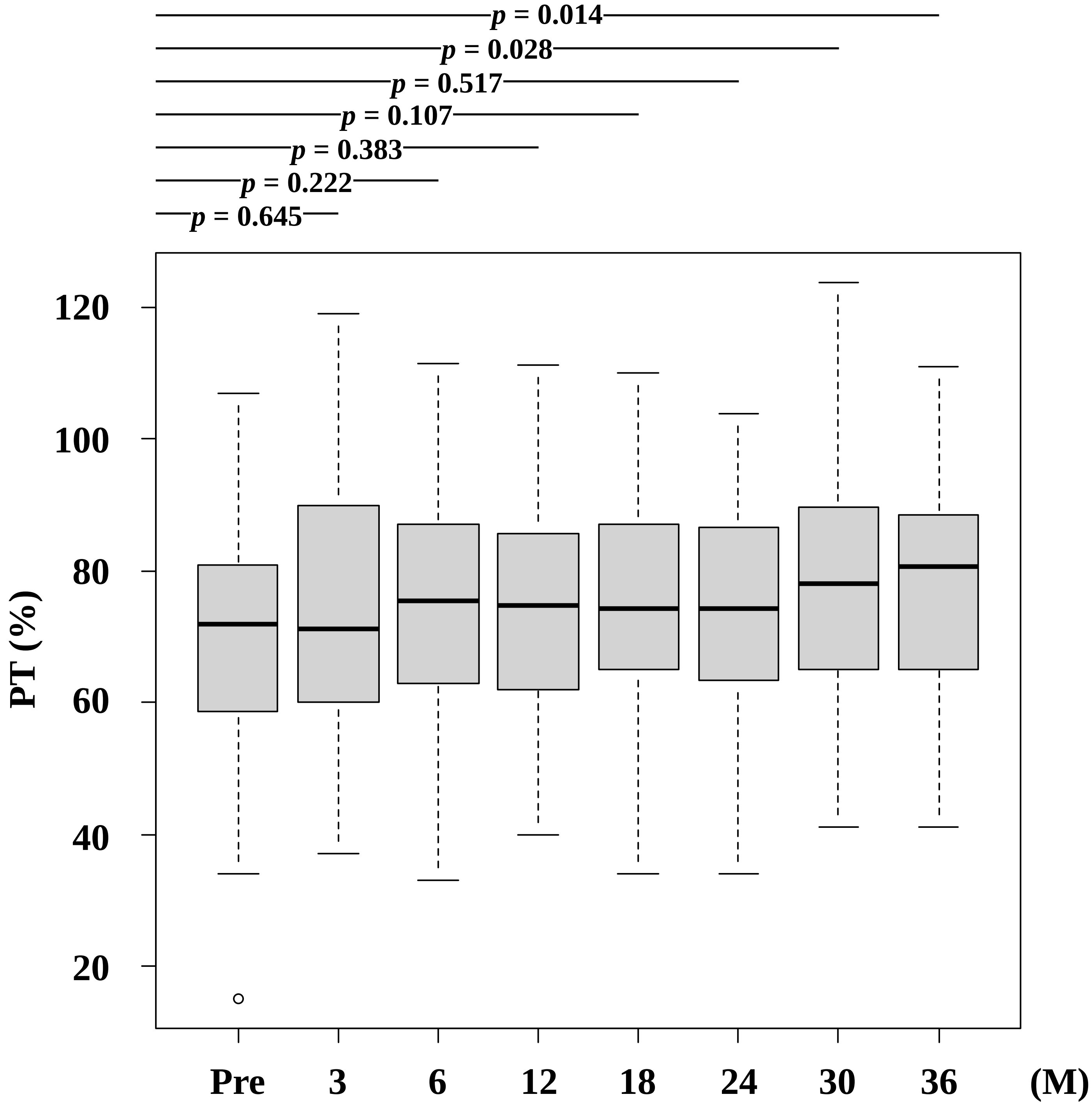

Figure S3d

≧65 years old

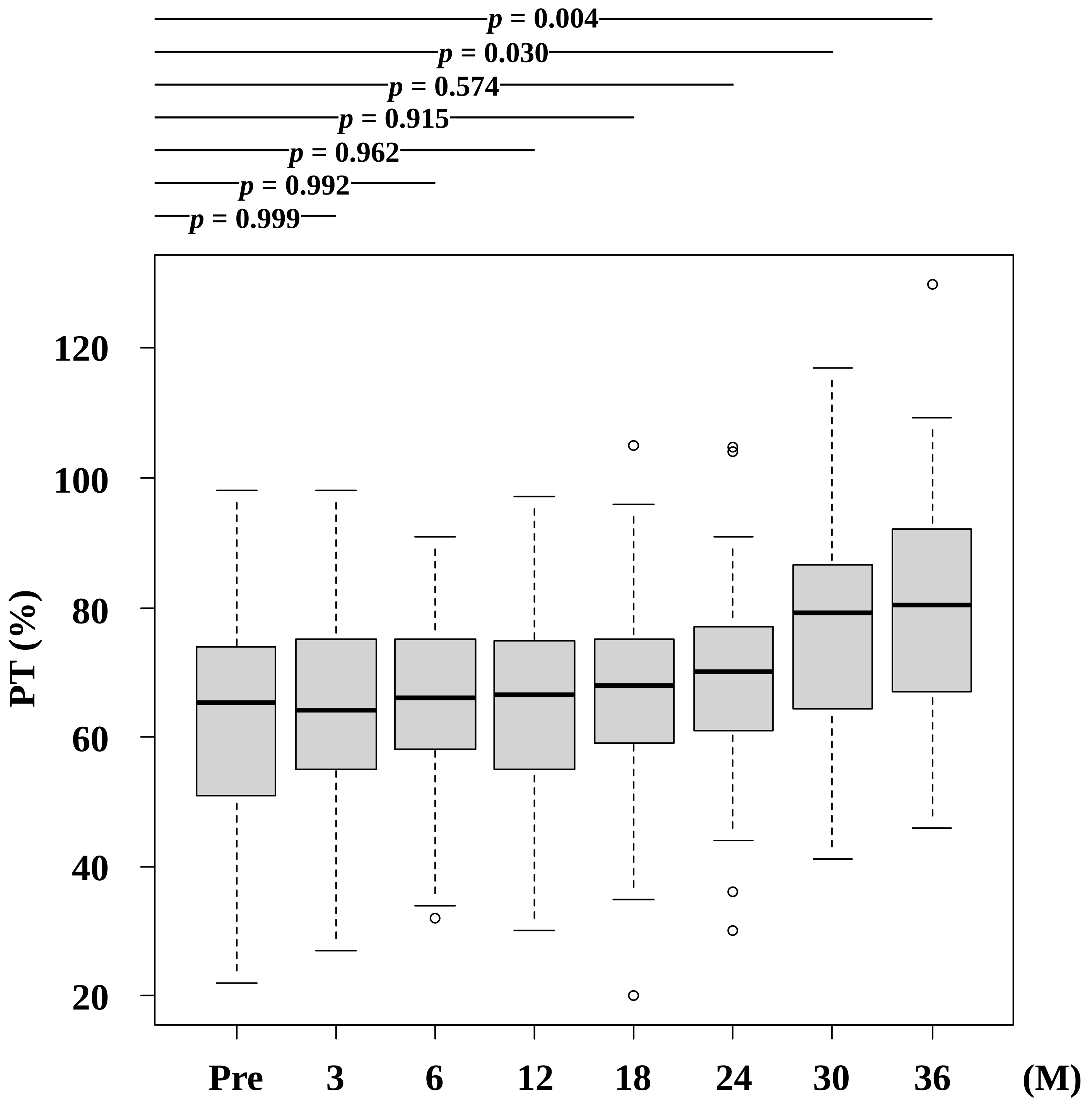

Figure S2e

Covert hepatic encephalopathy

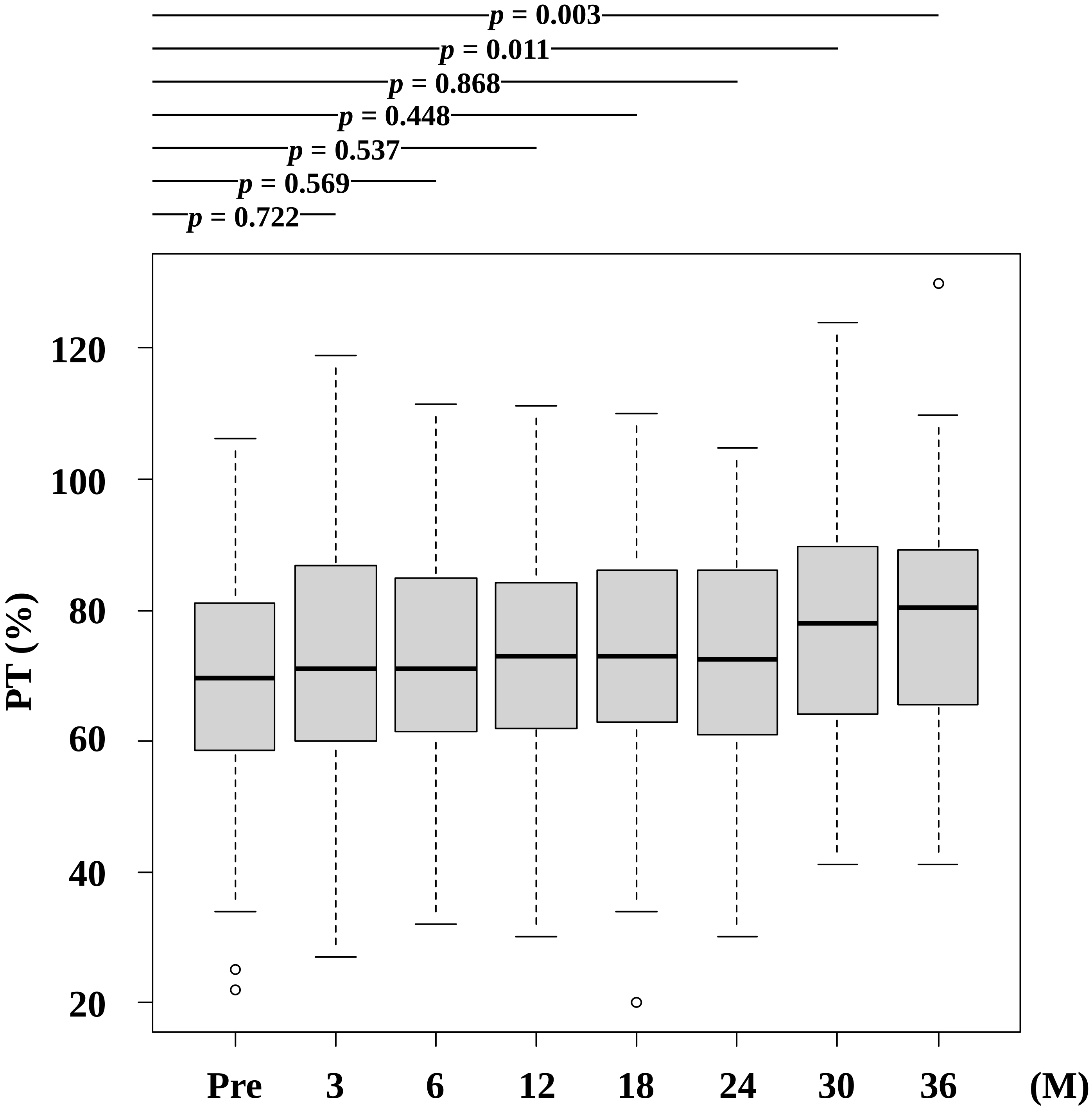

Figure S3f

Overt hepatic encephalopathy

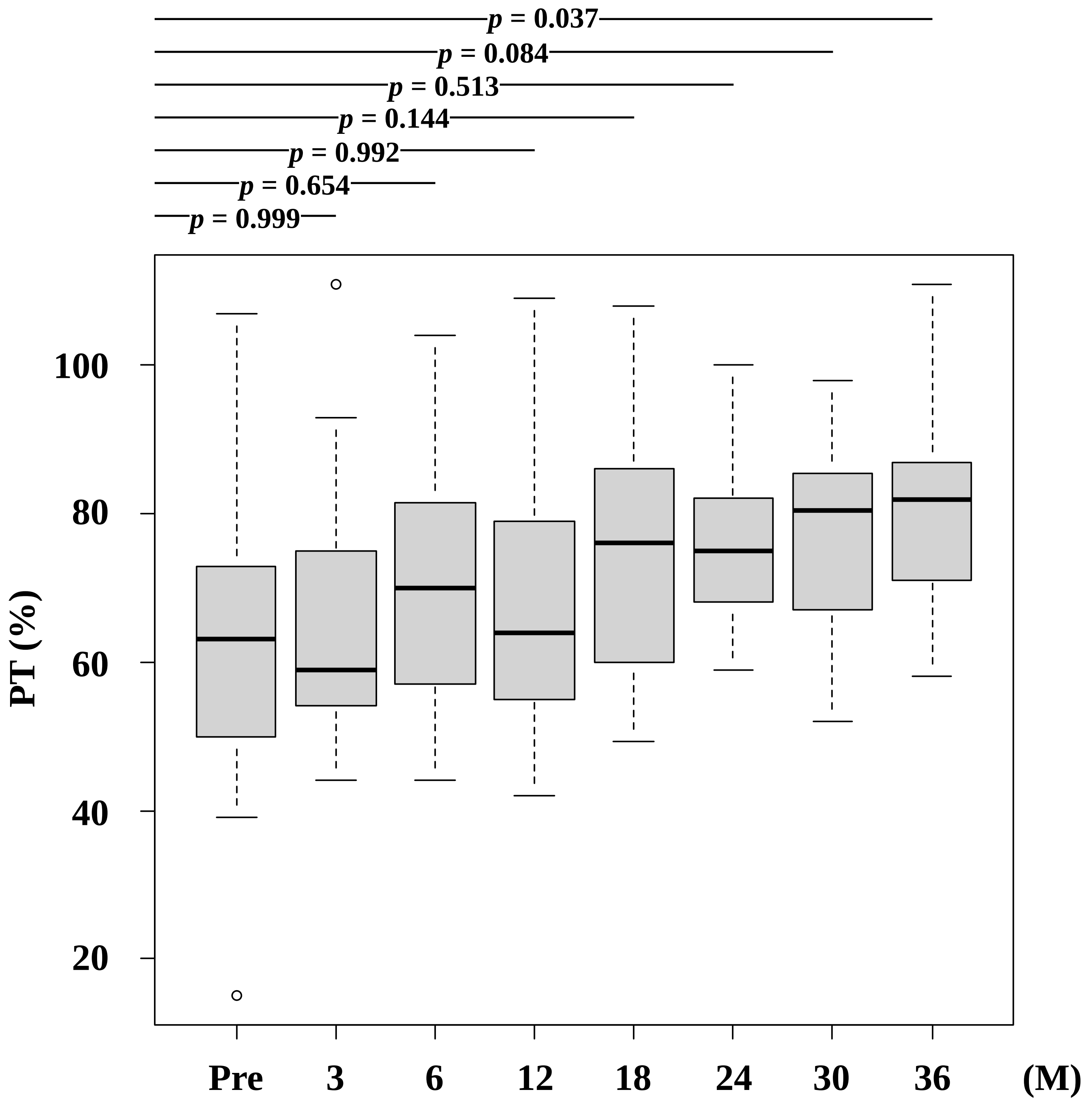

Supplement: Supplementary file 1 [file jcm-14-01358-s001.zip › jcm-3407159-supplementary.pdf]
